# Genome-wide association studies identify loci controlling specialized seed metabolites in Arabidopsis

**DOI:** 10.1093/plphys/kiad511

**Published:** 2023-09-27

**Authors:** Thomas Naake, Feng Zhu, Saleh Alseekh, Federico Scossa, Leonardo Perez de Souza, Monica Borghi, Yariv Brotman, Tetsuya Mori, Ryo Nakabayashi, Takayuki Tohge, Alisdair R Fernie

**Affiliations:** Central Metabolism, Max Planck Institute of Molecular Plant Physiology, Am Muehlenberg 1, 14476 Potsdam-Golm, Germany; Central Metabolism, Max Planck Institute of Molecular Plant Physiology, Am Muehlenberg 1, 14476 Potsdam-Golm, Germany; Central Metabolism, Max Planck Institute of Molecular Plant Physiology, Am Muehlenberg 1, 14476 Potsdam-Golm, Germany; Center of Plant Systems Biology and Biotechnology, 4000 Plovdiv, Bulgaria; Central Metabolism, Max Planck Institute of Molecular Plant Physiology, Am Muehlenberg 1, 14476 Potsdam-Golm, Germany; Research Center for Genomics and Bioinformatics (CREA-GB), Council for Agricultural Research and Economics, Via Ardeatina 546, 00178 Rome, Italy; Central Metabolism, Max Planck Institute of Molecular Plant Physiology, Am Muehlenberg 1, 14476 Potsdam-Golm, Germany; Central Metabolism, Max Planck Institute of Molecular Plant Physiology, Am Muehlenberg 1, 14476 Potsdam-Golm, Germany; Department of Biology, Utah State University, 5305 Old Main Hill, Logan, UT 84321-5305, USA; Department of Life Sciences, Ben-Gurion University of the Negev, 8410501 Be’er Sheva, Israel; RIKEN Center for Sustainable Resource Science, Tsurumi, 1-7-22 Suehiro, Yokohama, Kanagawa 230-0045, Japan; RIKEN Center for Sustainable Resource Science, Tsurumi, 1-7-22 Suehiro, Yokohama, Kanagawa 230-0045, Japan; Graduate School of Biological Science, Nara Institute of Science and Technology, 8916-5 Takayama-cho, Ikoma, Nara 630-0192, Japan; Central Metabolism, Max Planck Institute of Molecular Plant Physiology, Am Muehlenberg 1, 14476 Potsdam-Golm, Germany; Center of Plant Systems Biology and Biotechnology, 4000 Plovdiv, Bulgaria

## Abstract

Plants synthesize specialized metabolites to facilitate environmental and ecological interactions. During evolution, plants diversified in their potential to synthesize these metabolites. Quantitative differences in metabolite levels of natural Arabidopsis (*Arabidopsis thaliana*) accessions can be employed to unravel the genetic basis for metabolic traits using genome-wide association studies (GWAS). Here, we performed metabolic GWAS on seeds of a panel of 315 *A. thaliana* natural accessions, including the reference genotypes C24 and Col-0, for polar and semi-polar seed metabolites using untargeted ultra-performance liquid chromatography-mass spectrometry. As a complementary approach, we performed quantitative trait locus (QTL) mapping of near-isogenic introgression lines between C24 and Col-0 for specific seed specialized metabolites. Besides common QTL between seeds and leaves, GWAS revealed seed-specific QTL for specialized metabolites, indicating differences in the genetic architecture of seeds and leaves. In seeds, aliphatic methylsulfinylalkyl and methylthioalkyl glucosinolates associated with the *ALKENYL HYDROXYALKYL PRODUCING* loci (*GS-ALK* and *GS-OHP*) on chromosome 4 containing *alkenyl hydroxyalkyl producing 2* (*AOP2*) and *3* (*AOP3*) or with the *GS-ELONG* locus on chromosome 5 containing *methylthioalkyl malate synthase* (*MAM1*) and *MAM3*. We detected two unknown sulfur-containing compounds that were also mapped to these loci. In GWAS, some of the annotated flavonoids (kaempferol 3-*O*-rhamnoside-7-*O*-rhamnoside, quercetin 3-*O*-rhamnoside-7-*O*-rhamnoside) were mapped to *transparent testa 7* (*AT5G07990*), encoding a cytochrome P450 75B1 monooxygenase. Three additional mass signals corresponding to quercetin-containing flavonols were mapped to *UGT78D2* (*AT5G17050*). The association of the loci and associating metabolic features were functionally verified in knockdown mutant lines. By performing GWAS and QTL mapping, we were able to leverage variation of natural populations and parental lines to study seed specialized metabolism. The GWAS data set generated here is a high-quality resource that can be investigated in further studies.

## Teaser

Genome-wide association studies reveal loci controlling specialized seed metabolites in Arabidopsis, including glucosinolates, flavonoids, and unknown sulfur-containing compounds, providing a rich data set for future studies.

## Introduction

Two main phenotypic novelties have been critical during the transition from an aquatic to a terrestrial environment. One of these innovations was the emergence of phenylpropanoid and lignin biosynthesis, allowing early terrestrial plants to acquire a relatively rigid body structure and colonize the land (Weng and Chapple 2010). The other innovation consisted of the development of structures specialized for reproduction and dispersal, like pollen and seeds. These were essential for long-distance transport and successful colonization of the new environment by the offspring of primordial land plants (Linkies et al. 2010; Willis et al. 2014). Seeds, as a reproductive structure, also needed to be protected from adverse environmental conditions, including fungal attacks, insect feeding, or UV radiation. The chemical composition of seeds was thus selected not only to provide the essential nutrients during germination but also to accumulate a number of specialized metabolites conferring protective properties against biotic and abiotic stresses (Debeaujon et al. 2000).

Arabidopsis (*Arabidopsis thaliana*) is an ideal model for studying the link between phenotypic and genomic variation, given the wealth of available genomic resources (1001 Genomes Consortium, 2016 ; Togninalli et al. 2018). The considerable genetic variation of Arabidopsis was employed to study local adaptation in collections of natural accessions (Seren et al. 2017). GWAS is a technique to leverage natural variation and was used in previous studies to detect adaptive traits (Atwell et al. 2010; Togninalli et al. 2018). GWAS assesses the effect of each genomic marker at the population level, represented by information on high-density SNPs, on a quantitatively assessed phenotype with the likelihood of the association (Seren et al. 2017). QTL mapping, in comparison to GWAS, identifies genomic regions that co-segregate with a given trait in lines resulting from biparental or multiparental crosses. GWAS and QTL mapping were employed to study primary metabolism (Chan et al. 2010a; Wu et al. 2016; Slaten et al. 2020), specialized metabolism (Kliebenstein et al. 2001a; Hansen et al. 2008; Chan et al. 2010b; Chan et al. 2011; Routaboul et al. 2012; Li et al. 2014; Bac-Molenaar et al. 2015; Ishihara et al. 2016; Tohge et al. 2016; Wu et al. 2018), heavy metal (Chao et al. 2012) and salt tolerance (Baxter et al. 2010), shade avoidance (Filiault and Maloof 2012), and flowering time (Li et al. 2010).

In *A. thaliana*, two major classes of specialized metabolites have been considered to confer protective properties to abiotic stress, namely flavonoids and glucosinolates. Flavonoids are arguably the best-characterized class of specialized metabolites that are universally distributed in the plant kingdom (Winkel-Shirley 2001; Winkel-Shirley 2002; Falcone Ferreyra et al. 2012; Tohge et al. 2017). By analyzing flavonoid-less mutants (*banyuls* [*ban*], *apetala2* [*ap2*], and *transparent testa*), Debeaujon et al. (2000) could show that a lack of flavonoids resulted in lower dormancy and structural aberrations in seeds (missing layers, modified epidermal layers). Tepfer et al. (2012) and Tepfer and Leach (2017) showed that flavonoid-less seed mutants exhibited a lower survival rate when exposed to solar UV and cosmic radiation for 1.5 years. Generally, besides being involved in developmental and photoprotective processes, flavonoids convey antioxidative properties (Seyoum et al. 2006; Mierziak et al. 2014) and play a role in biotic stress (Treutter 2005; Mierziak et al. 2014); as yet, however, no such information is available if the same is true in seeds. In *A. thaliana* seeds, a wide array of flavonoids can be found, mainly belonging to the subclasses of flavonols (mono- and diglycosylated quercetin, kaempferol, and isorhamnetin derivatives) and of flavan-3-ols (epicatechin monomers and procyanidin polymers, Routaboul et al. 2006).

The other major class of specialized metabolites conferring tolerance to abiotic stress, glucosinolates, are mainly restricted to the Brassicales order, including the Brassicaceae, Capparaceae, and Caricaceae families but were also found in at least 500 noncruciferous angiosperm species (Fahey et al. 2001). The glucosinolate biosynthetic pathway and its regulation are well studied (Supplemental Fig. S1, Kliebenstein et al. 2001a, 2001b; Grubb and Abel 2006; Halkier and Gershenzon 2006; Hirai et al. 2007; Seo and Kim 2017). Glucosinolates are mainly attributed to being involved in biotic stress response (Grubb and Abel 2006; Halkier and Gershenzon 2006; Samuni-Blank et al. 2012). The role of glucosinolates in stress response was mainly defined through functional analysis of overexpression lines or mutants deficient in their regulation or biosynthesis (Beekwilder et al. 2008; Zhang et al. 2015). In *A. thaliana* seeds, 34 different glucosinolate species were detected that revealed different accumulation patterns in 39 different Arabidopsis ecotypes (Kliebenstein et al. 2001a). Two major glucosinolate subclasses, methylthioalkyl and methylsulfinylalkyl glucosinolates, showed striking differences between accessions: while the accessions Bs-1, Aa-0, Ma-0, and Yo-0 showed high methylthioalkyl:methylsulfinylalkyl glucosinolates ratio in seeds (>5), 13 accessions showed a ratio >3 (e.g., Sei-0, Tsu-1, and Mrk-0, Kliebenstein et al. 2001a). Furthermore, Kliebenstein et al. (2001a) found that glucosinolate accumulation differs between leaves and seeds: (i) the accessions Kas and Sorbo accumulate low levels of 2-hydroxy-3-butenyl glucosinolate in leaves, but high levels of this glucosinolate in seeds; (ii) the methylthioalkyl:methylsulfinylalkyl glucosinolates ratio in seeds is for all accessions >1, while for leaves this was only found in three accessions (Bla-10, Can-0, Su-0).

Previous studies in our group revealed differences in seed glucosinolate levels of *A. thaliana* Col-0, C24 (S. Alseekh, T. Tohge, A.R. Fernie, unpublished data) in introgression lines (Törjék et al. 2008). Taken together with previous findings that showed differences in the accumulation of seed specialized metabolites in Arabidopsis ecotypes, we conducted an untargeted metabolic profiling analysis by UPLC coupled to high-resolution mass spectrometry (MS) on *A. thaliana* seed polar and semi-polar metabolites (covering several classes of specialized metabolites) to reveal quantitative differences of metabolites between accessions. To find putative gene candidates that control the accumulation of specialized metabolites, we conducted GWAS and, in a complementary approach, QTL mapping on the Arabidopsis IL population obtained from the cross between C24 and Col-0. We show here that (i) previously characterized metabolites (flavonoids and glucosinolates) associate with known loci, (ii) two unknown sulfur-containing metabolites map to glucosinolates-associated loci, and (iii) that the respective Arabidopsis SALK knockdown lines of the gene *AT5G17050*, previously selected from GWAS, showed quantitative changes in the levels of the associated quercetin-containing flavonol compounds.

## Results and discussion

### Genome-wide association studies of untargeted seed metabolite analysis show a large set of mass feature pairs associated with the same loci

Genetic natural variation is an indispensable resource to find genes that are involved in the biosynthesis and regulation of plant specialized metabolites (Matsuda et al. 2015; Chen et al. 2016). Here, we determined the relative levels of polar and semi-polar seed metabolites from about 300 *A. thaliana* ecotypes using UPLC-MS from two growing seasons (replicate 1 and replicate 2) and from two previously published sets of leaf metabolites (Wu et al. 2018, Zhu et al. 2022), and mapped the features to their associated genomic loci using the same GWAS approach we applied previously (Zhu et al. 2022). This approach encompasses mixed linear models to account for the amount of phenotypic covariance caused by genetic relatedness, which should reduce confounding effects due to the population structure and kinship (Yu et al. 2006; Kang et al. 2008; Zhang et al. 2010; Vilhjálmsson and Nordborg 2013). Due to computational constraints, we did not identify epistatic interactions, even though these will contribute to the observed phenotypes (Marchini et al. 2005; Cordell 2009; Kam-Thong et al. 2011; Chen et al. 2014; Dong et al. 2015; Kerwin et al. 2015). Epistasis is the interaction of genetic variation at multiple loci that results in nonadditive effects in the analyzed phenotypes (Soltis and Kliebenstein 2015). In Arabidopsis, epistatic interactions typically involve the interaction of three or more loci (Wentzell et al. 2007; Rowe et al. 2008; Joseph et al. 2013a, 2013b; Kerwin et al. 2015).

To compare metabolites across the different sets, we matched the alignments of mass features of seed replicate 1, seed replicate 2/leaf_Wu_, and leaf_Zhu_ based on their *m*/*z* deviation, retention time deviation, and the covariance between the two seed replicates, resulting in 21,007 features for the negative and 36,194 features for the positive ionization mode. The leaf_Zhu_ data set was only available for the negative ionization mode. To further refine the accuracy, we imposed stricter matching rules, adjusting for retention time shift between replicate 1 and replicate 2 and a correlation of >0.1, resulting in a total number of 9,008 features for the negative and 12,133 features for positive ionization mode (core set). 2,882 (negative ionization mode) and 3,798 (positive ionization mode) matched mass features, i.e. those conserved between the aligned replication data sets, were mapped to the same locus/loci in GWAS (Supplemental Fig. S2). Those features that were mapped to the same locus/loci generally had higher heritability values (H^2^, Supplemental Fig. S2B, negative ionization mode: all: 0.549, mapped: 0.666; positive ionization mode: all: 0.542, mapped: 0.665) and higher correlation values (Supplemental Fig. S2A, negative ionization mode: all: 0.408, mapped: 0.534; positive ionization mode: all: 0.401, mapped: 0.529) than random pairs from the complete core set.

**Figure 1.**
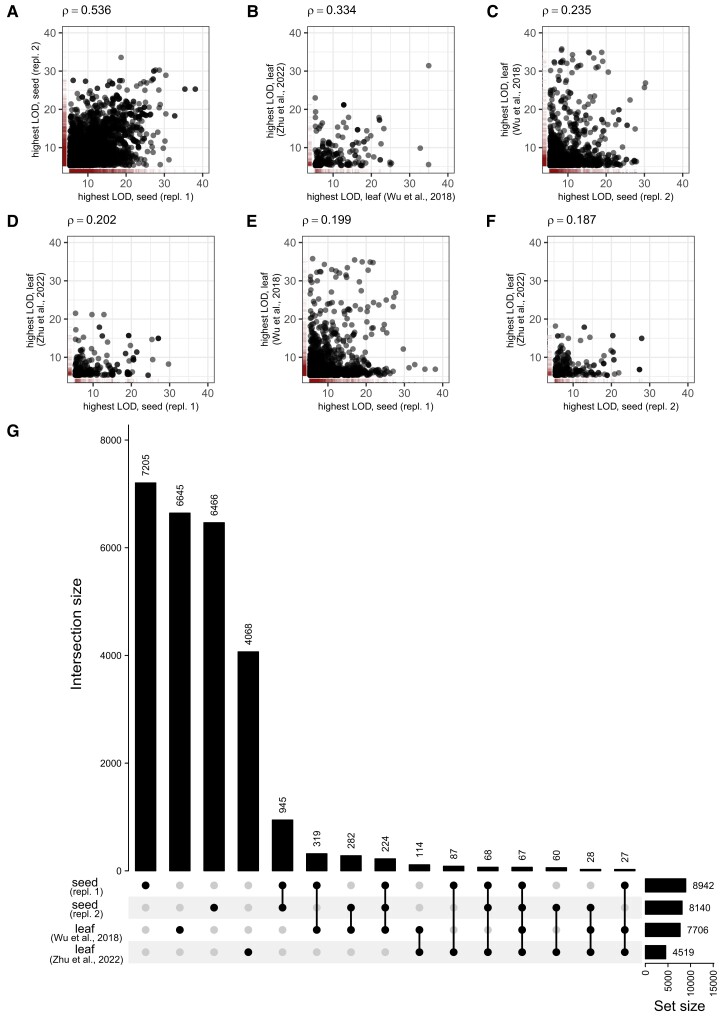
Mapping of seed and leaf replicates in genome-wide association studies (negative ionization mode). **A to F)** Scatterplots of highest LOD values of shared QTL per matched mass feature pair for the different data sets. The lanes display the density of data points. The Spearman correlation ρ values are indicated for the different data sets. **G)** Intersection sets of QTL for mass feature pairs with LOD ≥ 5.3. LOD, logarithm of odds; QTL, quantitative trait loci.

In the next step, we created a table with the GWAS results of the two biological replicates of seeds and the leaf samples. We reported for each joint mass feature the assigned QTL and LOD scores. From the core set, the two replicates from seed GWAS showed high positive Spearman correlation values for both data sets acquired in negative (ρ = 0.536) and positive (ρ = 0.557) ionization modes. The Spearman correlation values were lower (ρ between 0.187 and 0.291) when comparing the seed replicates with the result from the two leaf GWAS (Fig. 1, A to F, Supplemental Fig. S3, A and B). Furthermore, when looking at the intersection of shared loci (Fig. 1G, Supplemental Fig. S3D), we found that shared loci between the seed replicates showed a higher number (945 for negative and 4,085 for positive ionization mode) compared to that between seed and leaf GWAS (319/282/87/60 for negative and 2,405/1,620 for positive ionization mode). This may indicate that the reproducibility between the seed replicates of the core set is higher when compared to the results from the leaf GWAS. Alternatively, this may reflect a degree of variation in the genetic architecture. The comparison of the seeds and leaf data sets allowed the identification of tissue-specific QTL (Wu et al. 2018) and highlighted the different genetic architecture of these two tissues in controlling the accumulation of some specific mass features. However, there were 67 and 1,021 loci controlling the mass features in the core sets that are shared between the two seed replicates and the leaf data sets, indicating conserved loci controlling the levels of mass features across different tissues. We would like to point out that the leaf replicates showed fewer than expected similarities. We attributed this to differences in metabolite extraction, data acquisition and 1:many mappings in the alignment between the data sets, i.e. that we had for some mass features several features of the leaf_Zhu_ data set that matched to one feature in the seed and leaf_Wu_ data sets. This resulted in a considerably lower reported number of loci for the leaf_Zhu_ data set (Fig. 1G). The mass feature pairs that showed overlap between the two seed replicates, and for some of these also to the leaf data sets, represent a highly valuable resource that we make fully available in the Supplemental Data Sets S1 to S4. Shared loci between seed and leaf tissue represent reliable results of QTL along these variations in metabolism.

Alongside the shared loci, the majority of loci were not shared between the different mass feature sets (Fig. 1G and Supplemental Fig. S3D). When analyzing the distribution of the proportion of mass features mapped to loci, the sets that did not show intersection (seed replicate 1, seed replicate 2, leaf) had a higher proportion of two or more mass features mapped to the same loci compared to sets that show intersection (Supplemental Fig. S4). This could be attributed to measurement errors, associations of noncausative markers with a given trait, driven by linkage with causative markers (Korte and Farlow 2013), reflect environmental variance, or genotype–environment interaction effects. We assume that the significant associations from the intersection sets (Fig. 1G and Supplemental Fig. S3D), being conserved between the different replicates, represent genuine QTL characterized by lower sources of error. In the subsequent paragraphs, we only focus on those associations related to glucosinolates and flavanols, as well as on some unknown mass features supposedly representing glucosinolates and flavonoids in the seed data sets.

### Variation in glucosinolate levels in seeds is controlled by the *GS-ELONG*, *GS-ALK*, and *GS-OHP* loci

**Figure 2.**
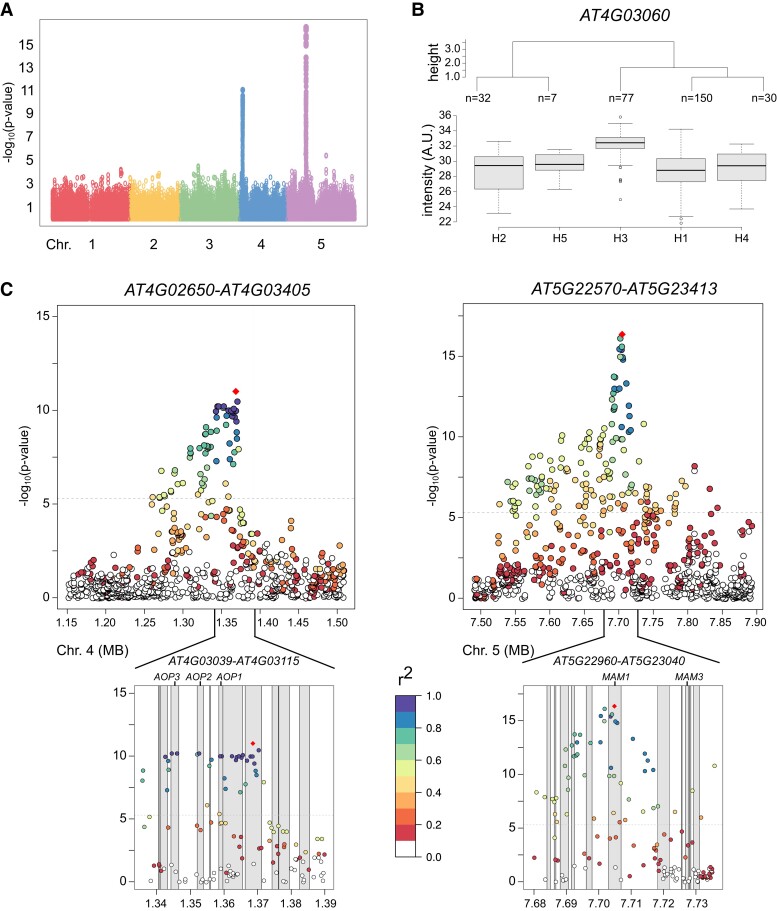
Genome-wide association mapping for 3-hydroxypropyl glucosinolate (seed, negative ionization mode). **A)** The Manhattan plot of 3-hydroxypropyl glucosinolate shows two peaks in each replicate on chromosomes 4 (highest LOD: 11.01) and 5 (16.35). These loci contain the genes *AOP1*, *AOP2*, and *AOP3* (chromosome 4), *MAM1* and *MAM3* (chromosome 5) that are involved in glucosinolate biosynthesis. Only seed replicate 1 is shown here. **B)** Haplotype analysis of metabolite levels of 3-hydroxypropyl glucosinolate. Shown are the log_2_-normalized intensities (A.U.) in boxplots (center line, median; lower and upper box limits, quartiles 1 and 3; whiskers, 1.5× interquartile range; points, outliers). The nucleotide sequence differences were statistically associated with the levels of 3-hydroxypropyl glucosinolate (ANOVA q-value: 1.78e−20 for replicate 1). Only data for seed replicate 1 is shown in A and B. The data for seed replicate 2 is depicted in Supplemental Fig. S5. **C)** LD analysis of the associated genomic regions on chromosomes 4 and 5 for 3-hydroxypropyl glucosinolate. The locus on chromosome 4 shows LD for the genomic region containing the genes *AOP1*, *AOP2*, and *AOP3*, while the locus on chromosome 5 marks a sharper decrease in standardized LD (*r*^2^), indicating that the *MAM1* gene is mainly responsible for the natural diversity of 3-hydroxypropyl glucosinolate levels. AOP, alkenyl hydroxyalkyl producing; A.U., arbitrary units; LD, linkage disequilibrium; LOD, logarithm of odds; MAM, methylthioalkylmalate synthase.

Based on previous studies, we annotated several metabolites in our data set, including amino acids, glucosinolates, and metabolites from the flavonoid and phenylpropanoid biosynthetic pathways (Supplemental Tables S1 and S2). The annotation of glucosinolates included all known methylsulfinylalkyl and methylthioalkyl glucosinolates. Most of these metabolites showed high broad-sense heritability (H^2^ > 0.75) and were mostly mapped with high LODs for both replicates to a locus on chromosome 5 and, for some of these glucosinolate metabolites, to a locus on chromosome 4 (Fig. 2A and Supplemental Fig. S1 A for 3-hydroxypropyl glucosinolate, Supplemental Tables S3 and S4). Within the locus on chromosome 5, the genes *methylthioalkylmalate synthase 1* (*MAM1*, *AT5G23010*) and *3* (*MAM3*, *AT5G23020*) are located, previously named the *GS-ELONG* locus. *MAM1* catalyzes the condensation reaction of two cycles of chain elongation in methionine-derived glucosinolate biosynthesis, and a *mam1* mutant showed a decrease in C_4_ and an increase in C_3_ glucosinolates (Kroymann et al. 2001). MAM3 accepts all ω-methylthio-2-oxoalkanoic acids required to synthesize C_5_ to C_8_ aliphatic glucosinolates in *A. thaliana* (Textor et al. 2007). Within the locus on chromosome 4, the genes *ALKENYL HYDROXYALKYL PRODUCING 1* (*AOP1, AT4G03070*), *AOP2* (*AT4G03060*), and *AOP3* (*AT4G03050*) are located, which are known as the *GS-ALK* and *GS-OHP* locus. The *AOP* genes encode 2-OG-dependent dioxygenases that are involved in glucosinolate biosynthesis. AOP2 and AOP3 convert methylsulfinylalkyl glucosinolates into alkenyl glucosinolates and hydroxyalkyl glucosinolates, respectively (Kliebenstein et al. 2001a, 2001b). In a study targeting specifically glucosinolate variation, Katz et al. (2022) also identified these loci regulating the glucosinolate chemotypes of *A. thaliana* seeds. It is important to note that the biochemical mechanisms by which modifications in these enzymes affect the content of these classes of glucosinolates are highly predictable on the basis of the enzymatic functions encoded by the genes and the position(s) occupied by the(se) enzyme(s) within the metabolic network (Chan et al. 2010b; Chan et al. 2011; Slaten et al. 2020). Indeed, some (although not all) of the candidates identified in our study were previously detected as QTL in previous studies (Chan et al. 2010b; Chan et al. 2011; Slaten et al. 2020). Examples of this are the identification of *MAM1*, *GS-ELONG*, and *AOP* loci in the previous works; however, it is important to note that since our study used a nontargeted approach of polar to semi-polar metabolites, we additionally provide a wider analysis than prior studies.

As complementation to the GWAS, we performed QTL mapping using biparental NILs from Col-0 and C24 (Törjék et al. 2008, Supplemental Data Set S5). This population was useful in detecting saiginols (phenylacylated flavonols) in floral tissues (Tohge et al. 2016). In the GWAS population and NIL population of Arabidopsis seeds, saiginols were not detected. The parental lines of the NIL population, C24 and Col-0, of the NIL population showed strong differences in relative glucosinolate levels: 4-methylsulfinylbutyl glucosinolate showed 8.40-times and 4-methylthiobutyl glucosinolate 5.05-times higher levels in Col-0 compared to C24; 3-butenyl glucosinolate showed 23.5-times and 8-methylsulfinyloctyl glucosinolate 265-times higher levels in C24 compared to Col-0. Subsequently, aliphatic glucosinolates showed strong relative metabolite differences in QTL mapping for genomic regions containing Col-0 *AOP* (Supplemental Fig. S6, Col-0 *AOP* in MASC05042-MASC09225 referring to the lines M36, M20, and M21).

Haplotypes of the genes *MAM1*, *MAM3*, and *AOP1*, *AOP2*, and *AOP3* showed significant differences in metabolite levels according to ANOVA (Fig. 2B and Supplemental Fig. S5B for 3-hydroxypropyl glucosinolate, GWAS population) indicating that the allelic variation at these target loci is responsible for the observed metabolite differences. Indeed, some of the SNPs for these genes involved in glucosinolate biosynthesis resulted in amino acid differences (Supplemental Table S5). The LD analysis for 3-hydroxypropyl glucosinolate revealed that the alleles on chromosome 4 are in high LD (standardized LD r^2^ close to 1) spanning the genomic region containing *AOP1*, *AOP2*, and *AOP3* (Fig. 2C, left panel). In *A. thaliana*, LD usually decays 50% within 5 kb (Gan et al. 2011; Korte and Farlow 2013). Here, the loci containing *AOP1*, *AOP2*, and *AOP3* showed wider LD. The situation on chromosome 5 marks a sharp decrease for 3-hydroxypropyl glucosinolate and peaks in the gene region of *MAM1* (*AT5G23010*). Interestingly, *MAM3* was in high LD (r^2^ > 0.6) with the SNP showing the highest LOD in *MAM1* but did not show as high *r*^2^ values as neighboring genes within the locus on chromosome 4. This indicates that *MAM1* is the main gene controlling 3-hydroxypropyl glucosinolate levels. Previously, these loci were also detected from GWAS of glucosinolate levels in leaves (Chan et al. 2011). The Arabidopsis *gtr1 gtr2* double mutant, which lacks (or contains low amounts, depending here on the type of the mutations it carries) the nitrate/peptide transporters responsible for glucosinolate transport to seeds, did not accumulate glucosinolates in seeds and exhibited a 10-fold over-accumulation in the source tissues leaves and silique walls (Nour-Eldin et al. 2012). Thus, it seems likely that the variation in glucosinolate levels is “inherited” from these source tissues.

### Unknown sulfur-containing metabolites map to *GS-ELONG*, *GS-ALK*, or *GS-OHP* loci in genome-wide association mapping

Next to the annotated glucosinolates, other mass features in the core set also showed an association with the *GS-ELONG*, *GS-ALK*, or *GS-OHP* loci in GWAS. In particular, two mass features with *m*/*z* of 596.1104 (unknown 596) and 626.1032 (unknown 626) were mapped to chromosome 4 or 5. The unknown 626 was mapped to the GS-ELONG locus (both seed replicates and leaf had a LOD ≥ 5.3 for the *GS-ELONG* locus, Supplemental Fig. S7), while the unknown 596 was mapped for both seed replicates to the GS-ALK and GS-OHP loci (LOD ≥ 5.3). Correlated mass features that showed *m*/*z* differences defined by the transformations (Supplemental Table S6) also showed associations with these loci (Supplemental Fig. S8).

The LD analysis revealed that for the unknown 626, the SNP with the highest LOD was located near or within the *MAM1* gene. The standardized LD, *r*^2^, decreased sharply when moving away from the *MAM1* gene (Supplemental Fig. S7). To reveal the chemical composition of the two unknowns, we fed isotope-labeled ^13^C and ^34^S to the siliques and analyzed the metabolites by LC-QTOF-MS. The unknown 596 (*m*/*z* 596.1104 in negative ionization mode) and 626 (*m*/*z* 626.1032 in negative ionization mode) contain most probably 22 C atoms and 23 C atoms, respectively, based on observation of shifts of *m*/*z* peaks in isotope feeding experiments with ^13^C. The MS analysis indicated for the feeding experiments with ^34^S that the two unknown compounds contain two and three S atoms (Supplemental Fig. S9). With estimation of chemical formula by accurate mass with number of carbons and sulfur atoms, these unknown peaks are annotated as sinapoyloxybutyl glucosinolate (C_22_H_31_NO_14_S_2_) and putative aromatic glucosinolate (C_23_H_33_NO_13_S_3_). Interestingly, in the biparental NIL population, the QTL mapping between C24 and Col-0 introgression lines of the unknowns 596 and 626 identified an additional locus close, but not overlapping, to *GS-ALK* and *GS-OHP* (*AT4G15733*-*AT4G24620*, M50_2_ in Supplemental Fig. S6). From the GWAS analysis here, the candidate region for the unknown 596 is *AT4G00005*-*AT4G03770*, overlapping with *AOP1*, *AOP2*, and *AOP3*. The introgression lines M20, M21, and M36, corresponding to the region *AT4G02465*-*AT4G08280*, showed higher levels of the unknowns 596 and 626 than the C24 background (Supplemental Fig. S6). Other glucosinolates, e.g. 8-methylsulfinyloctyl glucosinolate, 3-butenyl glucosinolate, and 4-methylsulfinylbutyl glucosinolate, did show elevated or decreased levels in these introgression lines (Supplemental Fig. S6A). Thus, it is unclear if the *GS-ELONG*, *GS-ALK*, and *GS-OHP* loci directly control the levels of the unknowns 596 and 626 or if, by an indirect effect, *AOP* and *MAM* change the flux of sulfur-containing metabolites. Alternatively, the genomic region from AT4G02465 to AT4G24620 may contain at least three glucosinolate-related loci for elevation and reduction of glucosinolate levels.

To narrow down candidate genes, in silico gene co-expression network analysis with known glucosinolate biosynthetic genes and genes located in the candidate genomic region (*AT4G02465*-*AT4G03770*) was performed (Supplemental Fig. S10). In the network component of known glucosinolate biosynthetic genes, *AOP2* (*AT4G03060*) and cysteine/histidine-rich C1 domain family protein gene (*AT4G02540*) are found. Further studies will be required in order to elucidate the precise mechanisms underlying these phenomena. The *AOP2* gene, which is the accession-specific 2-oxoglutarate-dependent dioxygenase gene and corresponds to the diversification of alkenyl glucosinolates among different ecotypes of Arabidopsis, is a potential candidate gene.

### Untargeted genome-wide association mapping of nonannotated mass features identifies a gene controlling flavonoid levels

**Figure 3.**
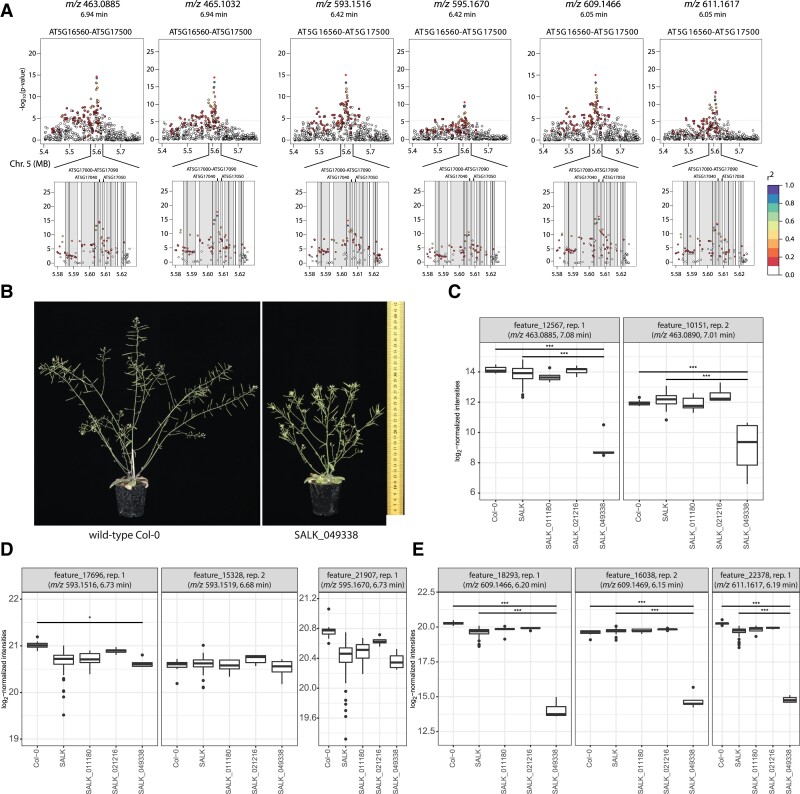
Flavonoid-biosynthetic pathway: mutant phenotype and functional analysis. **A)** Linkage disequilibrium analysis for quercetin-containing flavonols (negative and positive ionization mode). The highest LOD is achieved for a SNP within the region of *AT5G17030* or *AT5G17040*. Standardized LD *r*^2^ is relatively low for the SNPs that are located within the gene *AT5G17050*. Only data for seed replicate 1 are shown in **(A)**. The data for seed replicate 2 are depicted in Supplemental Fig. S11. **B)** The mutant of *AT5G17050* (SALK_049338) exhibited a dwarf phenotype with a loss of apical dominance (stunted inflorescence), as reported previously by Yin et al. (2014). The scale bar is valid for all images shown here. Images were digitally extracted for comparison. Additional biological replicates are shown in Supplemental Fig. S13. **C to E**) To test for seed metabolite differences between the mutant lines and Col-0 or the other SALK lines, we performed moderated *t*-tests. Shown are the log_2_-normalized intensities (A.U.) in boxplots (center line, median; lower and upper box limits, quartiles 1 and 3; whiskers, 1.5× interquartile range; points, outliers). Metabolite analysis of mapped mass features *m*/*z* 463.0885/465.1032, *m*/*z* 593.1516/595.1670, and *m*/*z* 609.1466/611.1617 (negative/positive ionization mode) showed lower levels in the seeds of mutant lines (*n* = 5 individual plants) compared to wild-type Col-0 (*n* = 9). A.U., arbitrary units. LD, linkage disequilibrium; LOD, logarithm of odds; MB, megabase; SNP, single nucleotide polymorphism. *P-value < 0.05, **P-value < 0.01, ***P-value < 0.001.

The mass features with *m*/*z* 463.0885/465.1032 (7.08 min), 593.1516/595.1670 (6.73 min), 609.1466/611.1617 (6.20 min, negative/positive ionization mode, *m*/*z* values and retention time from the UPLC-MS analysis of SALK lines), and co-eluting mass features were mapped to the genes *AT5G17040* and *AT5G17050* (*UGT78D2*, Fig. 3A and Supplemental Fig. S11) in GWAS. Given the characteristic *m*/*z* of 303.0504 and other spectrometric peaks (positive ionization mode), these metabolites were putatively annotated as quercetin hexoside (*m*/*z* 463.0885/465.1032), quercetin deoxyhexoside deoxyhexoside (*m*/*z* 593.1516/595.1670), and quercetin hexoside deoxyhexoside (*m*/*z* 609.1466/611.1617). The chromatographic peaks were distinct from other flavonols with the same *m*/*z*, e.g. quercetin 3-*O*-rhamnoside-7-*O*-rhamnoside (retention time of replicate 1: 6.71 min, unknown *m*/*z* 593.1516/595.1670: 6.43 min).

To validate the associations, we selected genes of interest based on (i) the LOD score from GWAS, (ii) the expression of the gene from the data reported by Schmid et al. (2005) (Affymetrix ATH1 array), (iii) haplotype and LD analysis, and (iv) potential involvement of the gene in the biosynthetic pathway based on homology analysis and literature support for quercetin-containing flavonols and other nonannotated mass features. For the flavonol-related metabolites, we selected two genes of interest; for unknown mass features in our core set, we selected seven genes of interest and obtained T-DNA insertion SALK lines for functional validation. We provide a list of the SALK T-DNA lines in Supplemental Table S7. Except for three SALK lines, which showed to be heterozygous for the insertion, homozygosity was confirmed by PCR genotyping in the T2 generation (Supplemental Table S7), and SALK lines were individually cultivated in two replicates. Heterozygous lines might be critical if the underlying trait shows complete dominance and the associated metabolite levels do not differ between wild-type and mutant lines. In case of incomplete dominance, the effect might not be detectable by a statistical test due to low effect size and low statistical power. Seeds of the SALK lines were analyzed by UPLC-MS (Supplemental Fig. S12). The resulting data set was analyzed in terms of the presence and differential abundance of the associated mass features via moderated t-tests with respect to Col-0 and other SALK line seeds in negative and positive ionization modes. Only the line SALK_049338 (*AT5G17050*, encoding UDP-GLUCOSYL TRANSFERASE 78D2) showed differential abundance for several mass features compared to the control lines (Supplemental Tables S8 and S9).

When growing the line SALK_049338 (*AT5G17050*), we observed shorter stature for all plants as compared to the wild-type (Fig. 3B and Supplemental Fig. S13), a phenotype also previously reported when mutating this gene (Yin et al. 2014). Yin et al. (2014) suggested that a *ugt78d2* mutant causes reduced polar auxin transport in shoots via alterations in the levels of kaempferol 3-*O*-rhamnoside-7-*O*-rhamnoside, which is likely responsible for the shorter stature. The quercetin-containing flavonols *m*/*z* 463.0885/465.1032 and *m*/*z* 609.1466/611.1617 in this line exhibited lower seed metabolite levels compared to the other SALK lines (excluding the lines for *AT5G17040* and *AT5G17050*) and wild-type Col-0, while levels of *m*/*z* 593.1516/595.1670 were not affected by *AT5G17050* (Fig. 3, C to E).

Flavonoids are involved in the regulation of auxin transport (Buer and Muday 2004; Peer and Murphy 2007). Lee et al. (2005) and Tohge et al. (2005) described that UGT78D2 is a flavonoid 3-*O*-glucosyltransferase and that *ugt78d2* mutants show an altered flavonoid pattern. A *ugt78d1* (*AT1G30530*) *ugt78d2* double mutant exhibited strong and specific repression of flavonol biosynthesis and was strongly impaired in the initial 3-*O*-glycosylation, while UGT78D3 (AT5G17030) only contributed to a minor extent to overall 3-*O*-glycosylation (Jones et al. 2003; Tohge et al. 2005; Yonekura-Sakakibara et al. 2008; Yin et al. 2012).

UGT73C6 (AT2G36790) is the 7-*O*-glucosyltransferase expressed in flowers; however, 7-*O*-rhamnosylation by UGT89C1 (AT1G06000) is more common as the form of 7-*O*-conjugation (Yonekura-Sakakibara et al. 2008). Yin et al. (2014), studying *UGT78D2*, suggested that kaempferol 3-*O*-rhamnoside-7-*O*-rhamnoside is responsible for the altered growth phenotype by narrowing down the potential active moieties using a series of mutants. In the same study, a *ugt78d1 ugt78d2* double mutant showed strongly reduced levels of kaempferol 3-*O*-glucoside-7-*O*-rhamnoside and kaempferol 3-*O*-[rhamnosyl (1→2 glucoside)]-7-*O*-rhamnoside, while kaempferol 3-*O*-rhamnoside-7-*O*-rhamnoside was not detected at all. Furthermore, the levels of the aglycones kaempferol and quercetin were reduced to 21% and 18% of the wild-type levels, respectively.

Interestingly, the unknown quercetin deoxyhexoside deoxyhexoside (*m*/*z* 593.1516/595.1670), presumably containing rhamnoside, did not show lower levels in the *ugt78d2* mutant lines, despite the fact that the unknown flavonol showed an association with *UGT78D2* in GWAS. This could be explained by the fact that *UGT78D2* is a glucosyltransferase, not a rhamnosyltransferase (Yin et al. 2014), and could indicate that *UGT78D2* indirectly controls the flux of rhamnosylated (deoxyhexosylated) flavonols in seeds. In our GWAS data set, kaempferol 3-*O*-rhamnoside-7-*O*-rhamnoside (H^2^ = 0.837 in positive ionization mode) showed association with a gene in the region *AT5G01680*-*AT5G13170* but not with the locus containing *AT5G17050* (Supplemental Table S4). The SNP with the highest LOD (≥7.8 in positive ionization mode) located close to *transparent testa 7* (*tt7*, *AT5G07990*, Supplemental Data Set 2). TT7 is a cytochrome P450 75B1 monooxygenase, an enzyme previously reported to have 3′-flavonoid hydroxylase activity (Schoenbohm et al. 2000) that regulates the kaempferol/quercetin ratio (Peer et al. 2001). Similarly, quercetin 3-*O*-rhamnoside-7-*O*-rhamnoside (H^2^ = 0.921 in positive ionization mode) was mapped with a LOD ≥ 6.2 close to *TT7* (Supplemental Data Set 2, positive ionization mode). On the other hand, kaempferol 3-*O*-glucoside-7-*O*-rhamnoside (H^2^ = 0.173 in positive ionization mode) had its highest LOD within the gene *UGT78D3* (replicate 2, no mapping with LOD ≥ 5.3 for replicate 1). For the other annotated flavonol glycosides (in positive ionization mode) in the core set, no genome-wide association was obtained. For QTL mapping, no associations with flavonoids were detected. This is to be expected since annotated flavonoid levels in the biparental lines showed little differences: kaempferol 3-*O*-glucoside-7-*O*-rhamnoside showed 1.24-times, quercetin 3-*O*-glucoside-7-*O*-rhamnoside 1.10-times, kaempferol 3-*O*-rhamnoside 1.26-times, and quercetin 3-*O*-rhamnoside 1.16-times higher levels in C24 compared to Col-0 (Supplemental Fig. S6). For GWAS, missing associations could be due to low absolute variation of these metabolites or because these flavonoids are regulated by multiple loci that are not reported as significant in our approach. Higher differences in accumulation patterns can be triggered through the application of different kinds of stress (e.g. UV radiation) before analyzing metabolite levels. This finding was generally in line with a previous smaller-scale study that detected quantitative rather than qualitative differences in flavonoids between *A. thaliana* accessions and concluded that most flavonoids are controlled by a few additive loci with relatively broad effects (Routaboul et al. 2012).

Here, we focused on the analysis of the association involving candidate structural genes. In this paper, we focused on candidate structural genes of the glucosinolate/flavonoid pathways, although we report in the Supplemental Data Set 1 to S5 the full list of significant associations from GWAS and QTL mapping that may represent a resource to investigate the additional control these metabolites may have at the level of pathway regulation. Furthermore, the results from glucosinolates and some of the flavonoids indicated pleiotropic effects and collocating QTL for joint mass features. This analysis can be extended to a wider scale and to nonbiosynthetic enzymes. Moreover, the core set exhibited differences in QTL between the mass features from the two seed replicates and the leaf replicate. Future studies will investigate the variation in the genetic architecture of traits controlling the levels of specialized metabolites across different tissues.

## Conclusions

Here, we performed GWAS on metabolic mass features of two biologically independent replicates of seed from two growing seasons and one replicate of leaves obtained by untargeted UPLC-MS. As a complementary approach, we performed QTL mapping of NIL introgression lines between C24 and Col-0 for specific seed metabolites. By including GWAS of leaf metabolites, we detected 4,884 and 5,688 loci for mass feature pairs (negative and positive ionization mode) that were exclusively detected for seed GWAS, indicating differences in tissue-specific associations between seeds and leaves. On the other hand, 1,026 and 1,247 QTL for mass feature pairs (negative and positive ionization mode) were conserved across seed and leaf tissues in GWAS.

In seeds, aliphatic methylsulfinylalkyl and methylthioalkyl glucosinolates, as well as two unknown sulfur-containing compounds, tentatively identified as previously uncharacterized glucosinolates, showed associations in GWAS and QTL mapping with the known *GS-ELONG*, *GS-ALK*, or *GS-OHP* loci. In addition, QTL mapping detected an adjacent region on chromosome 4 for the two unknown sulfur-containing compounds. In GWAS, some of the annotated flavonoids in seeds showed associations with regions containing *TT7* or *UGT78D2*, including three previously unknown quercetin-containing flavonols. QTL mapping did not reveal any association for flavonoids. This difference is potentially caused by the low allelic variance in flavonoid-biosynthetic genes, resulting in small differences in flavonoid levels in the parental lines.

A SALK knockdown line of the gene *UGT78D2* (*AT5G17050*) showed decreased levels of the quercetin-containing flavonols, while SALK lines of the neighboring gene *AT5G17040* did not show changes in flavonol levels. We would like to draw the following conclusions regarding the genetic architecture of seed specialized metabolism: (*i*) seed specialized metabolism differs substantially from leaf metabolism as shown by the identification of QTL that differ between these tissues, but the two tissues also exhibit common genetics to some degree; (*ii*) *AOP* and *MAM* genes are key regulators for glucosinolate seed metabolite levels in seeds. Aliphatic glucosinolates are presumably not synthesized in situ in seeds but are transported from source tissues to seeds. The variation of aliphatic glucosinolates is “inherited” from these source tissues; (*iii*) the alleles of *UGT78D2* (*AT5G17050*) affect the levels of quercetin-containing flavonols in seeds. The natural GWAS population was shaped by processes of genetic adaptation and meiotic events during evolution. This results in greater phenotypic variance compared to the NIL population between Col-0 and C24, as exemplified by differences in flavonoid levels. However, the overlap suggests, as previously stated (Brog et al. 2019), that genome-wide association and QTL mapping are complementary techniques to study seed specialized metabolism.

## Materials and methods

### Plant material

The HapMap collection of natural Arabidopsis (*A. thaliana*) accessions (315 accessions) with existing SNP data (Li et al. 2010; Horton et al. 2012) was used to perform GWAS on polar and semi-polar metabolites. Seed material for GWAS analysis was provided by Yariv Brotman (MPI-MP, Potsdam, Germany) and grown by the Green team of the Max Planck Institute of Molecular Plant Physiology in two growing seasons in the years 2017 (replicate 1) and 2018 (replicate 2) according to Wu et al. (2018). Seeds were sown directly to soil in 6 cm pots for each accession and stratified in a growth chamber (Percival Scientific, Perry, USA; 250 μE m^−2^ s^−1^ day/night 16 h/8 h, temperature of 20 °C/6 °C, relative humidity, RH, 60%/75%). After two weeks (end of March), the seedlings were pricked and transferred to separate pots with six replicates per accession. Plants were randomly placed in a polytunnel greenhouse (with an integrated frost protection system) and randomly dislocated every 1 to 2 weeks to avoid positional shading. Plants were bagged one month before harvest for seed collection (glassine bags, 40 g m^−2^). Two weeks before harvest, watering was stopped. Plants were harvested from the end of May until the middle of June, depending on the genotype. Harvested bagged inflorescences were stored for four weeks at 15 °C and 15% RH. Seeds were collected from these six replicates per accession by sieving siliques (sieve size 355, Edinger Direkt, Leinburg, Germany) into glass vials before storing them at 15 °C, 15% RH. These seeds were used for the seed GWAS. Leaf samples for GWAS analysis were obtained by Wu et al. (2018) using the control condition samples.

The introgression line population of Arabidopsis (near-isogenic lines, NILs), obtained from the cross between Col-0 and C24 (Törjék et al. 2008), was cultivated as described in Tohge et al. (2016). Seeds were collected from three individual plants of 45 m lines (C24 background) and 69 N lines (Col-0 background), as described above. These seeds were used for the seed QTL analysis.

The SALK lines (SALK_008908, SALK_011180, SALK_020876, SALK_021216, SALK_024438, SALK_027837, SALK_037430, SALK_049338, SALK_072964, SALK_081021, SALK_201809C, SALK_203337C, SALK_203919C, SALK_204674C, SALK_206494C) were obtained from the NASC database. C24, Col-0, and SALK mutant lines were cultivated under greenhouse conditions (21/19 °C, day/night 16/8 h, RH 50%/50%, additional illumination by Philips Son-T Agro lamps from 6 Am-10 Am and 6 Pm-10 Pm; Philips, Eindhoven, The Netherlands). The plants of the different lines were randomly placed to avoid block effects during growth. Plants were watered daily with 1/1000 Hyponex solution (Hyponex, Osaka, Japan). The trays with plants were randomly distributed two times per week to prevent positional light effects. Seeds were collected as described above. We provide a list with information on the SALK mutant lines in Supplemental Table S7.

The leaf material for the GWAS analysis of leaf_Wu_ and leaf_Zhu_ was taken directly from Wu et al. (2018) and Zhu et al. (2022), respectively. While polar to semi-polar were extracted and measured for the leaf_Wu_ data set as described below, for the leaf_Zhu_ the mass feature table was directly taken from Zhu et al. (2022) and GWAS was performed based on this table.

### Genotyping of Col-0 and SALK lines

About 4 weeks after germination, one leaf per replicate (in total five replicates) was collected from Col-0, C24, and SALK lines, frozen in liquid N_2_, and stored at −80 °C. DNA was extracted according to (Kasajima et al. 2004). Col-0 wild-type plants and the SALK lines were genotyped by PCR using the following mix: 15.7 *μ*L water, 2 *μ*L 10× DreamTaq buffer, 0.4 *μ*L 10 mm dNTP, 0.4 *μ*L LBb1.3 or line-specific forward primer, 0.4 *μ*L line-specific reverse primer, 0.1 *μ*L DreamTaq polymerase (Thermo Fisher Scientific, Waltham, USA), 1 *μ*L template DNA. The primers are described in Supplemental Table S10. The following program was used (Biometra T Professional Thermocycler, Analytik Jena, Jena, Germany): 5 min initial denaturation, 95 °C; 35 cycles of 30 s denaturation, 95 °C, 30 s annealing 58 °C, 1 min extension, 72 °C; 10 min final extension, 72 °C; hold, 4 °C. 20 *μ*L of PCR product was separated on a 1% [w/v] agarose gel for 25 min at 120 V.

### Extraction of polar and semi-polar metabolites in seeds and leaves

Metabolites from seeds were extracted according to (Tohge and Fernie 2010). 200 *μ*L of pre-cooled (−20 °C) 80% [v/v] MeOH (Sigma-Aldrich, Munich, Germany; containing 1 *µ*g isovitexin and 0.04 mg ribitol as internal standard) was added to 30 *A. thaliana* seeds (cooled in liquid N_2_), of which the weight was previously determined. After shaking the tubes, previously cooled in liquid N_2_ (3 min, 25 *Hz* by Retsch mill MM 301, Haan, Germany), the tubes were centrifuged for 10 min at room temperature (17,900 *g*), and the supernatant was transferred to a new tube. The tubes were centrifuged for 10 min at room temperature (17,900 *g*). 135 *μ*L of the supernatant were transferred to a new tube, dried by speed-vac for 2 to 3 h, filled with argon, and stored at −80 °C. On the day of analyses, the samples were resuspended in 100 *μ*L 80% [v/v] MeOH and transferred to sample vials.

Metabolites from leaves for the leaf_Wu_ data set were extracted from 50 mg leaf material (cooled in liquid N_2_) using 500 *μ*L of the same extraction buffer as above. The same extraction protocol was followed as above, transferring 200 *μ*L of the supernatant to a new tube before drying by speed-vac for 2 to 3 h. On the day of analyses, the samples were resuspended in 200 *μ*L 80% [v/v] MeOH and transferred to sample vials.

### Determination of relative polar and semi-polar metabolite levels by UPLC-MS for genome-wide association studies for seeds and leaf_Wu_

For leaf and seed metabolites, extracts from Col-0, prepared as described above, were taken as a quality control. Metabolites were separated by Waters Acquity UPLC I using a Waters Acquity UPLC BEH C18 1.7 *μ*m VanGuard^TM^ 2.1 × 5 mm as a pre-column and a Waters Acquity UPLC HSS T3 1.8 *μ*m 2.1 × 100 mm as a column (Waters, Dublin, Ireland; injection volume 5 *μ*L, sample temperature 10 °C, column temperature 40 °C, flow rate 0.4 mL min^−1^). The gradient was as follows: from 0 to 1 min 99% buffer A (Water UL/C MS grade (Bio-Lab ltd., Jerusalem, Israel) + 0.1% [v/v] formic acid) and 1% buffer B (acetonitrile UL/C MS grade (Bio-Lab ltd., Jerusalem, Israel) + 0.1% [v/v] formic acid), 11 min 60% A and 40% B, 13 min 30% A and 70% B, 15 min 1% A and 99% B isocratic flow to 16 min, 17 min 99% A and 1% B isocratic flow to 20 min. Metabolites were ionized by ESI in negative and positive ionization modes (capillary voltage ±3.5 kV, sheath gas flow 60, auxiliary gas flow 20, capillary temperature 275 °C, drying gas temperature 300 °C, skimmer voltage 25 V, tube lens voltage 130 V). MS spectra were acquired from 1 to 20 min by Thermo Scientific Q Exactive in Full MS mode (resolution 70000, max. injection time 100 ms, automatic gain control value 3E6; Thermo Fisher Scientific, Waltham, USA) in the scan range 100 to 1500  *m*/*z*. Peaks per replicate and ionization mode were aligned by Genedata (version 10.5.3) using the settings according to Supplemental Table S11. Mass features that eluted before 0.5 min and after 16 min were removed from the peak alignment. For each ionization mode separately, the replicates were combined by matching based on a *m*/*z* deviance of ±0.01 and a retention time deviance of ±0.3 min to obtain the joint mass features present in both replicates. Intensity values were divided by the respective analyzed seed weight. Intensity values were log_2_ transformed, and batch effects were removed by the function removeBatchEffect from the limma package (v3.38.3, Ritchie et al. 2015). In the case of multiple matches from replicate 1 to replicate 2, only the matched feature pairs with the highest covariance were retained. Outliers were removed by checking their intensity values by boxplots and by projecting them via principal component analysis (PCA) by the function prcomp} from the stats package (v.4.1.2) in R.

### Determination of relative polar and semi-polar seed metabolite levels by HPLC-MS and QTL mapping

Metabolite levels were determined according to Tohge et al. (2016) using an HPLC system Surveyor (high pressure LC; Thermo Finnigan, Waltham, USA) coupled to a Finnigan LTQ-XP system (Thermo Finnigan, Waltham USA). Chromatographic data were processed via Xcalibur (v2.1, Thermo Fisher Scientific, Waltham, USA). QTL mapping was done according to Tohge et al. (2016).

### ^13^C and ^34^S isotope feeding and measurement by LC-quadrupole time-of-flight (QTOF) MS

*A. thaliana* seeds were labeled with ^13^C (via ^13^CO_2_) and ^34^S (via Na_2_^34^SO_4_) according to Nakabayashi et al. (2013) and Nakabayashi et al. (2016) using Col-0 plants prepared by SI Science Co., Ltd. (Saitama, Japan). The dried samples were extracted with 150 *μ*l for ^13^C samples and 50 *μ*l for ^34^S of 80% [v/v] MeOH containing 2.5 *μ*M 10-c amphour sulfonic acid per mg dry weight using a mixer mill with zirconia beads for 7 min at 18 Hz and 4 C. After centrifugation for 10 min, the supernatant was filtered using an HLB μElution plate (Waters). The extracts (1 *μ*l) were analyzed using LC-QTOF-MS (LC, Waters Acquity UPLC system; MS, Waters Xevo G2 Q-Tof). Analytical conditions were as follows LC: column, Acquity bridged ethyl hybrid (BEH) C18 (1.7 *μ*m, 2.1 mm 100 mm, Waters); solvent system, solvent A (water including 0.1% [v/v] formic acid) and solvent B (acetonitrile including 0.1% [v/v] formic acid); gradient program, 99.5%A/0.5%B at 0 min, 99.5%A/0.5%B at 0.1 min, 20%A/80%B at 10 min, 0.5%A/99.5%B at 10.1 min, 0.5%A/99.5%B at 12.0 min, 99.5%A/0.5%B at 12.1 min and 99.5%A/0.5%B at 15.0 min; flow rate, 0.3 ml/min at 0 min, 0.3 ml/min at 10 min, 0.4 ml/min at 10.1 min, 0.4 ml/min at 14.4 min and 0.3 ml/min at 14.5 min; column temperature, 40 C; MS detection: polarity, negative; capillary voltage, −2.75 kV; cone voltage, 25.0 V; source temperature, 120 C; desolvation temperature, 450 C; cone gas flow, 50 l/h; desolvation gas flow, 800 l/h; collision energy, 6 V; mass range, *m*/*z* 50 to 1500; scan duration, 0.1 s; interscan delay, 0.014 s; data acquisition, centroid mode; Lockspray (Leucine enkephalin); scan duration, 1.0 s; interscan delay, 0.1 s.

### Determination of relative polar and semi-polar metabolite levels by UPLC-MS for Col-0, C24, and SALK mutant lines

Metabolites were separated by Waters Acquity UPLC using a Waters HSS T3 C18 (Waters, Dublin, Ireland, 100 mm I. × 2.1 mm i.d. × 1.8 *μ*m particle size) as column and pre-column (column temperature 40 °C, flow rate 0.4 mL min^−1^). The gradient was as follows: 1 min 99% buffer A (Water UPLC-MS grade + 0.1% [v/v] formic acid; Biosolve, Dieuze, France) and 1% buffer B (acetonitrile + 0.1% [v/v] formic acid; Biosolve, Dieuze, France), 11 min 60% A and 40 B, 13 min 30% A and 70% B, 15 min 1% A and 99% B isocratic flow to 16 min, 17 min 99% A and 1% B isocratic flow to 20 min. Metabolites were ionized by ESI in negative and positive ionization modes (capillary voltage ±3 kV, sheath gas flow 60, auxiliary gas flow 35, capillary temperature 150 °C, drying gas temperature 350 °C, skimmer voltage 25 V, tube lens voltage 130 V). MS spectra were acquired from 1 to 19 min by ThermoScientific Q Exactive in MS mode (resolution 25000, max. injection time 100 ms, automatic gain control value 1E6; Thermo Fisher Scientific, Waltham, USA) in the scan range 100 to 1500 *m*/*z*. Peaks were aligned by xcms (v3.16.1, Smith et al. 2006) and annotated by CAMERA (v.1.50.0, Kuhl et al. 2012) in the R programming language (v4.1.2, see Supplemental Table S12). Intensity values were divided by the respective seed weight. Intensity values were log_2_ transformed, and batch effects were removed by the function removeBatchEffect from the limma package (v3.38.3). Outliers were removed by checking their quality via the MatrixQCvis package (v1.5.4, Naake and Huber 2022). Metabolite and mass features were checked by the Thermo Xcalibur Qual Browser (v4.0.27.21, Thermo Fisher Scientific, Waltham, USA).

### Genome-wide association mapping, calculation of heritability, haplotype and linkage disequilibrium analysis, and statistical testing for differences in SALK lines

A similar approach to Fusari et al. (2017) and Wu et al. (2018) was taken to map metabolite information to genetic loci. The R packages EMMAX (Efficient Mixed-Model Association eXpedited, Kang et al. 2010) and GAPIT (Genomic Association and Prediction Integrated Tool, version 23-May-18, Lipka et al. 2012) were used to perform the mapping. We employed a mixed linear model containing fixed and random effects and characterized the population structure using the first three principal components (Q matrix, Price et al. 2006) to incorporate this information together with the VanRaden kinship matrix (Eu-Ahsunthornwattana et al. 2014) as fixed and random effects, respectively (method = “MLM”). The aligned mass feature table with normalized intensity values was used as an input. The GAPIT function was used to map the phenotypic observations (normalized metabolite intensities) to loci in the *A. thaliana* genome using 199,455 SNP markers with minor allele frequency > 1% obtained using Affymetrix GeneChip Array 6.0 (TAIR version 9, Li et al. 2010; Horton et al. 2012) using PCA.total = 3, model = “MLM”, SNP.fraction = 1.0 (all other parameters were set to default). Information on the genes/locus tags of TAIR9 can be in Supplemental Table S13. The logarithm of odds (LOD) threshold was set to 5.3 (−log_10_(1/*N*) with *N* the number of SNPs). The resulting SNPs with LOD ≥ 5.3 were assigned to the same group if the genomic distance between them was less than 10 kb, and the genes within the respective groups were considered as candidate genes.

Broad-sense heritability (H^2^) was defined by the proportion of the total variance explained by the genetic variance according to Fusari et al. (2017) using the lmer function and obtaining the variances by the function VarCorr from lme4 (v1.1–23, Bates et al. 2015). For calculating the heritability, only the features were used that showed a retention time deviance of ≤0.075 min (retention time_repl.1_—retention time_repl.2_), an absolute *m*/*z* deviance of ≤0.075, and a Pearson correlation of >0.1.

For haplotype analysis, the distance between haplotypes was calculated from the SNPs by the dist.gene function from the ape (v5.3, Paradis and Schliep 2019) package (method = “pairwise”, pairwise.deletion = FALSE, variance = FALSE). Distances were clustered by the hclust function (method = “ward.D”), and the tree was cut by cutree (h = 0.00001) from the stats package (v3.6.2). To test for statistical relation between haplotypes and metabolite levels, ANOVA (anova from the stats package, v3.6.2) was performed with FDR correction (false discovery rate, p.adjust with method = “BH”), adjusting for the number of all metabolites used for mapping in negative and positive ionization mode. For linkage disequilibrium (LD) analysis, the *P*-values were taken from the GWAS results file for the respective mass feature (LOD = -log_10_(*P*-value)). Standardized LD, r^2^, values were calculated via the function r2fast from the GenABEL package (v1.8-0, Aulchenko et al. 2007). Expression analysis for genes of interest was conducted within the eFP browser (Winter et al. 2007) using the data set of Schmid et al. (2005).

To test for differences in SALK lines, the log2-normalized raw intensities were tested against Col-0 or the respective complement of SALK lines using limma (v3.50.3). To this end, linear models were fitted for each metabolic feature using lmFit and moderated t-statistics were computed by empirical Bayes moderation of the standard errors towards a global value using eBayes (trend = TRUE). *P*-values were adjusted using FDR via the Benjamini–Hochberg method. Since there was no corresponding second replicate available in positive ionization mode, the corresponding features in negative ionization mode were determined using correlation analysis and retention time window thresholding. If multiple features in negative ionization mode matched the feature in positive ionization mode, the feature with the highest correlation to the feature of positive mode (replicate 1) was selected.

The scripts can be found at https://www.github.com/tnaake/GWAS_arabidopsis_seed.

### MetNet network construction

*m*/*z* and retention time values of seed replicate 1 were used for structural network inference via structural and rtCorrection from the MetNet package (v1.15.3, R v4.1.2, Naake and Fernie 2019) using the transformations and retention time shifts described in Supplemental Table S6. Edges corresponding to adduct additions were removed if the retention time between two mass features was >0.1 min. The combined peaklists with log-normalized intensity values of replicate 1 and 2 were used as input for statistical network construction (function statistical) using Pearson and Spearman correlation. The weighted statistical adjacency matrices were thresholded (function threshold), only retaining correlation values >0.7 for Pearson and Spearman correlation coefficients and FDR-adjusted *P*-values < 0.05 using the Benjamini–Hochberg method. The network was visualized in Cytoscape (v3.7.2, Shannon et al. 2003). The script can be found at https://www.github.com/tnaake/GWAS_arabidopsis_seed.

### Accession numbers

Sequence data for the major genes mentioned in this article can be found in the GenBank library under accession numbers NM_104992 (AT1G63140), KJ138817 (AT3G21750), NM_113074 (AT3G21790), NM_001340416 (AT4G03050), BT029463 (AT4G03060), NM_116541 (AT4G03070), NM_202795 (AT4G09500), NM_117019 (AT4G09510), NM_119903 (AT4G37400), NM_119904 (AT4G37410), BT000794 (AT4G37430), NM_001343478 (AT5G17040), AY128739 (AT5G17050), NM_001343784 (AT5G23010), NM_122208 (AT5G23020).

## Supplementary Material

kiad511_Supplementary_Data

## Data Availability

All processed data is provided in the Supplementary Datasets associated with the article.

## Supplemental data

The following materials are available in the online version of this article.

**Supplemental Figure S1.** Simplified pathway for aliphatic glucosinolates.

**Supplemental Figure S2.** Metabolite data sets for seed replicates 1 and 2 (genome-wide association studies).

**Supplemental Figure S3.** Mapping of seed replicates 1 and 2 and leaf_Wu_ in genome-wide association studies (positive ionization mode).

**Supplemental Figure S4.** Distribution of number of mass features mapped to locus/loci per intersection set.

**Supplemental Figure S5.** Genome-wide association mapping for 3-hydroxypropyl glucosinolate (seed, negative ionization mode).

**Supplemental Figure S6.** QTL mapping using near-isogenic introgression lines between C24 and Col-0 seeds (C24 background).

**Supplemental Figure S7.** Manhattan plot and linkage disequilibrium analysis of the unknown 626 (seed, negative ionization mode).

**Supplemental Figure S8.** Seed metabolite network of mapped mass features (negative ionization mode).

**Supplemental Figure S9.** Effects of ^13^C and ^34^S feeding on the *m*/*z* of the unknowns 596 and 626 (negative ionization mode).

**Supplemental Figure S10.** Gene co-expression network analysis of glucosinolate biosynthetic genes and genes in the candidate genomics region.

**Supplemental Figure S11.** Flavonoid-biosynthetic pathway: Linkage disequilibrium analysis for quercetin-containing flavonols (seed, negative, and positive ionization mode).

**Supplemental Figure S12.** Principal component analysis for seed metabolite analysis of SALK lines (replicate 1).

**Supplemental Figure S13.** Phenotype of Col-0 and SALK_049338 mutant lines.

**Supplemental Table S1.** Annotated metabolites in negative ionization mode.

**Supplemental Table S2.** Annotated metabolites in positive ionization mode.

**Supplemental Table S3.** Quantitative trait loci for annotated metabolites for negative ionization mode.

**Supplemental Table S4.** Quantitative trait loci for annotated metabolites for positive ionization mode.

**Supplemental Table S5.** Amino acid change for genes of interest.

**Supplemental Table S6.** Transformations for MetNet.

**Supplemental Table S7.** SALK lines for functional characterization of genes.

**Supplemental Table S8.** Change in mass feature levels in SALK mutant lines (negative ionization mode).

**Supplemental Table S9.** Change in mass feature levels in SALK mutant lines (positive ionization mode).

**Supplemental Table S10.** Primers for SALK lines.

**Supplemental Table S11.** Parameters for Genedata alignment.

**Supplemental Table S12.** Parameters for xcms/CAMERA alignment.

**Supplemental Table S13.** Information on genes/locus_tag of TAIR9.

**Supplemental Table S14.** RNAseq profiling of 10 time points during germination in Arabidopsis, from freshly harvested seed, through mature seed, stratification, germination, and to postgermination.

**Supplemental Data Set 1.** Loci from GWAS for seed replicate 1, seed replicate 2, and leaf_Wu_ (negative ionization mode).

**Supplemental Data Set 2.** Loci from GWAS for seed replicate 1, seed replicate 2, and leaf_Wu_ (negative ionization mode).

**Supplemental Data Set 3.** Loci from GWAS for seed replicate 1, seed replicate 2, leaf_Wu_, and leaf_Zhu_ (negative ionization mode).

**Supplemental Data Set 4.** Peak tables for seed relicate 1, seed replicate 2, leaf_Wu_, and leaf_Zhu_ (negative and positive ionization mode).

**Supplemental Data Set 5.** Seed QTL mapping of introgression line population.

## Dive Curated Terms

The following phenotypic, genotypic, and functional terms are of significance to the work described in this paper:

At5g17050 Gramene: AT5G17050
At5g17050 Araport: AT5G17050

UGT78D2 Gramene: AT5G17050
UGT78D2 Araport: AT5G17050

aop1 Gramene: AT4G03070
aop1 Araport: AT4G03070

AT5G17040 Gramene: AT5G17040
AT5G17040 Araport: AT5G17040

AT4G03770 Gramene: AT4G03770
AT4G03770 Araport: AT4G03770

TT7 Gramene: AT5G07990
TT7 Araport: AT5G07990

AT1G30530 Gramene: AT1G30530
AT1G30530 Araport: AT1G30530

AT4G02540 Gramene: AT4G02540
AT4G02540 Araport: AT4G02540

AT4G08280 Gramene: AT4G08280
AT4G08280 Araport: AT4G08280

AT4G24620 Gramene: AT4G24620
AT4G24620 Araport: AT4G24620
