# Supplementary material for: Genome-wide association studies identify loci controlling specialized seed metabolites in Arabidopsis
Source: Plant Physiol. 2023 Sep 27;194(3):1705–21. doi: 10.1093/plphys/kiad511 (PMC10904349; doi:10.1093/plphys/kiad511)
Supplement: kiad511_Supplementary_Data [file kiad511_supplementary_data.zip › SupplementalData Thomas Naake.pdf]

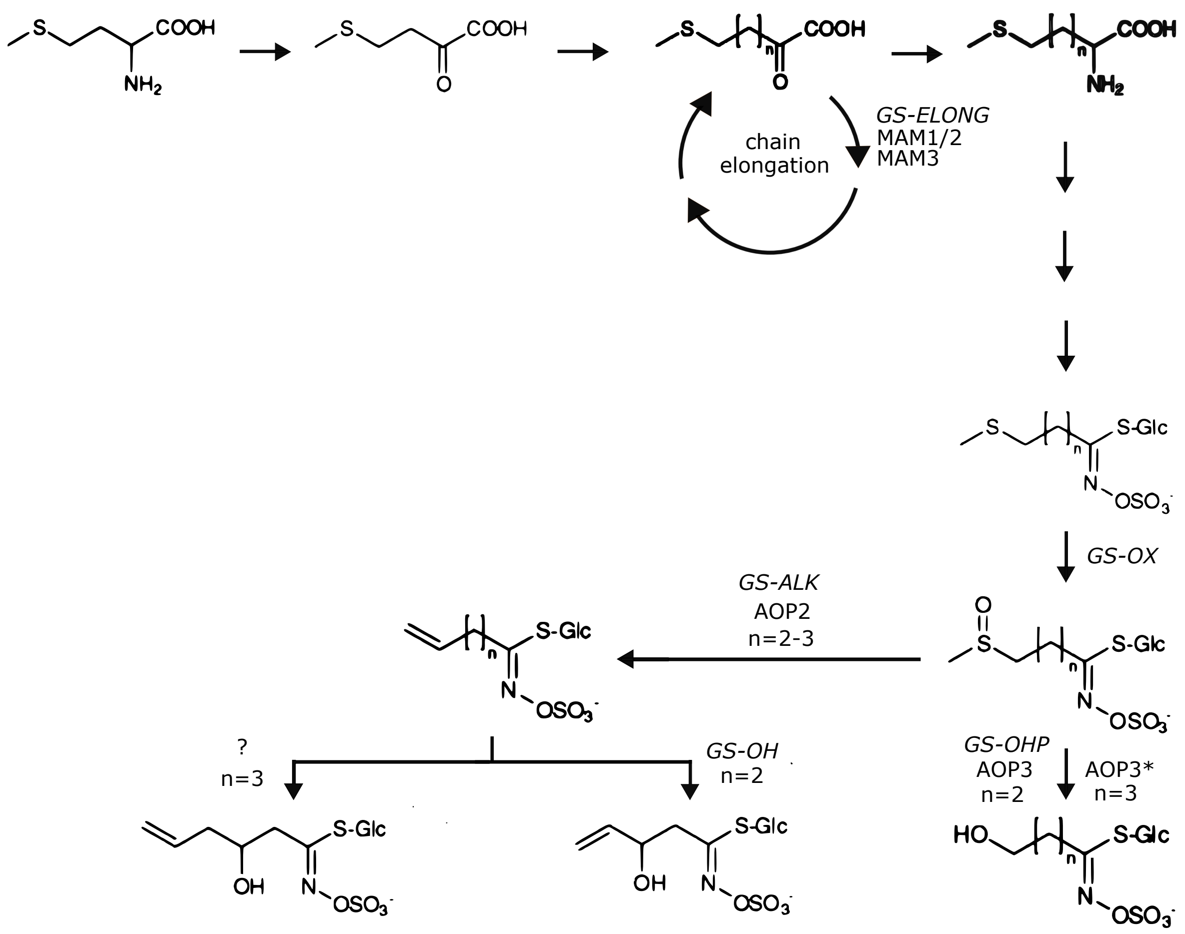

Supplemental Figure S1: Simplified pathway for aliphatic glucosinolates. The biosynthetic pathway starts with the deamination of methionine to a 2-oxo acid by a branched-chain amino acid aminotransferase. Subsequently, the 2-oxo acid enters a cycle of three successive transformations: condensation with acetyl-CoA by MAM, isomerization by an isopropylmalate isomerase, and oxidative decarboxylation by an isopropylmalate dehydrogenase (Sønderby et al., 2010). One round of chain elongation leads to an elongation of one ethylene group (-CH_2-). After several intermediate steps, including sulfur incorporation, the side chain of aliphatic glucosinolates is modified by *GS-OX*, *GS-ALK*, *GS-OH*, and *GS-OHP* (Sønderby et al., 2010). AOP: alkenyl hydroxyalkyl producing; MAM: methylthioalkylmalate synthase; *: predicted enzyme; ?: unknown enzyme.

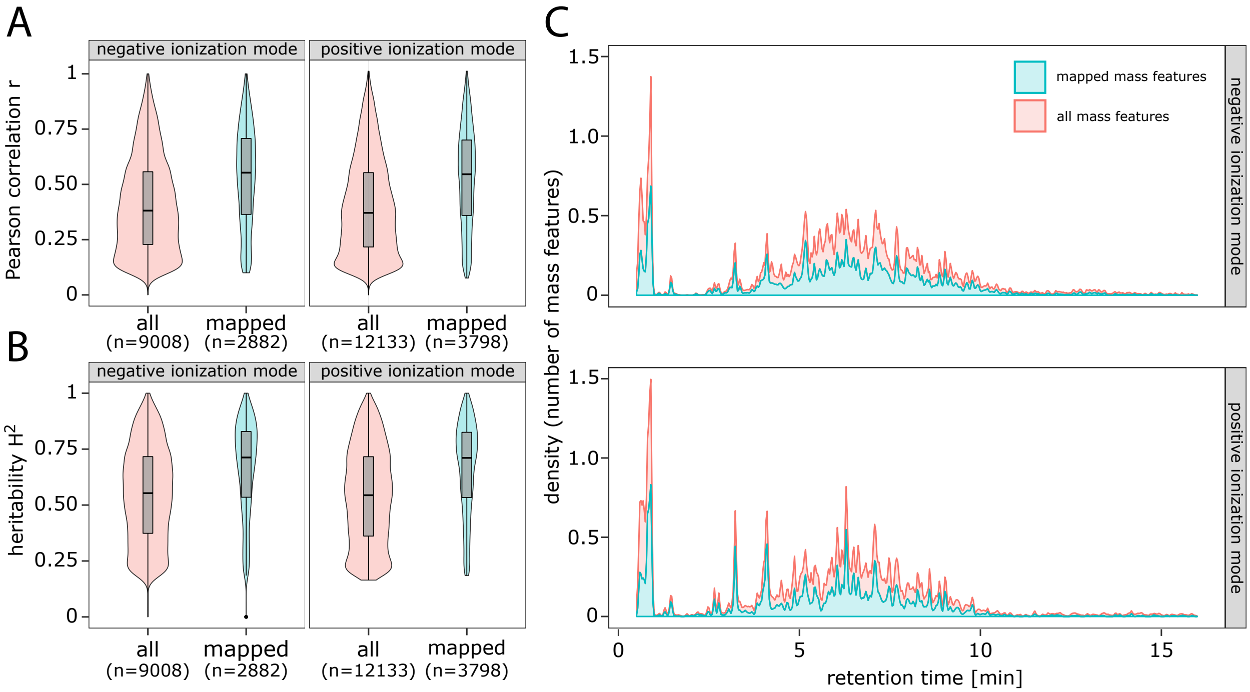

Supplemental Figure S2: Metabolite datasets for seed replicates 1 and 2 (genome-wide association studies). A: Pearson correlation coefficient (r) values of intensity values for matched mass feature pairs for negative and positive ionisation mode. r values of mapped mass features were higher compared to the random pairs of the core set. B: Broad-sense heritability (H^2) of intensity values for matched mass feature pairs for negative and positive ionisation mode. H^2 values of mapped mass features were higher compared to the random pairs of the core set. C: Distribution along retention time for all matched mass feature pairs and those that were mapped to at least one locus with LOD ≥ 5.3 for negative and positive ionisation mode. LOD: logarithm of odds.

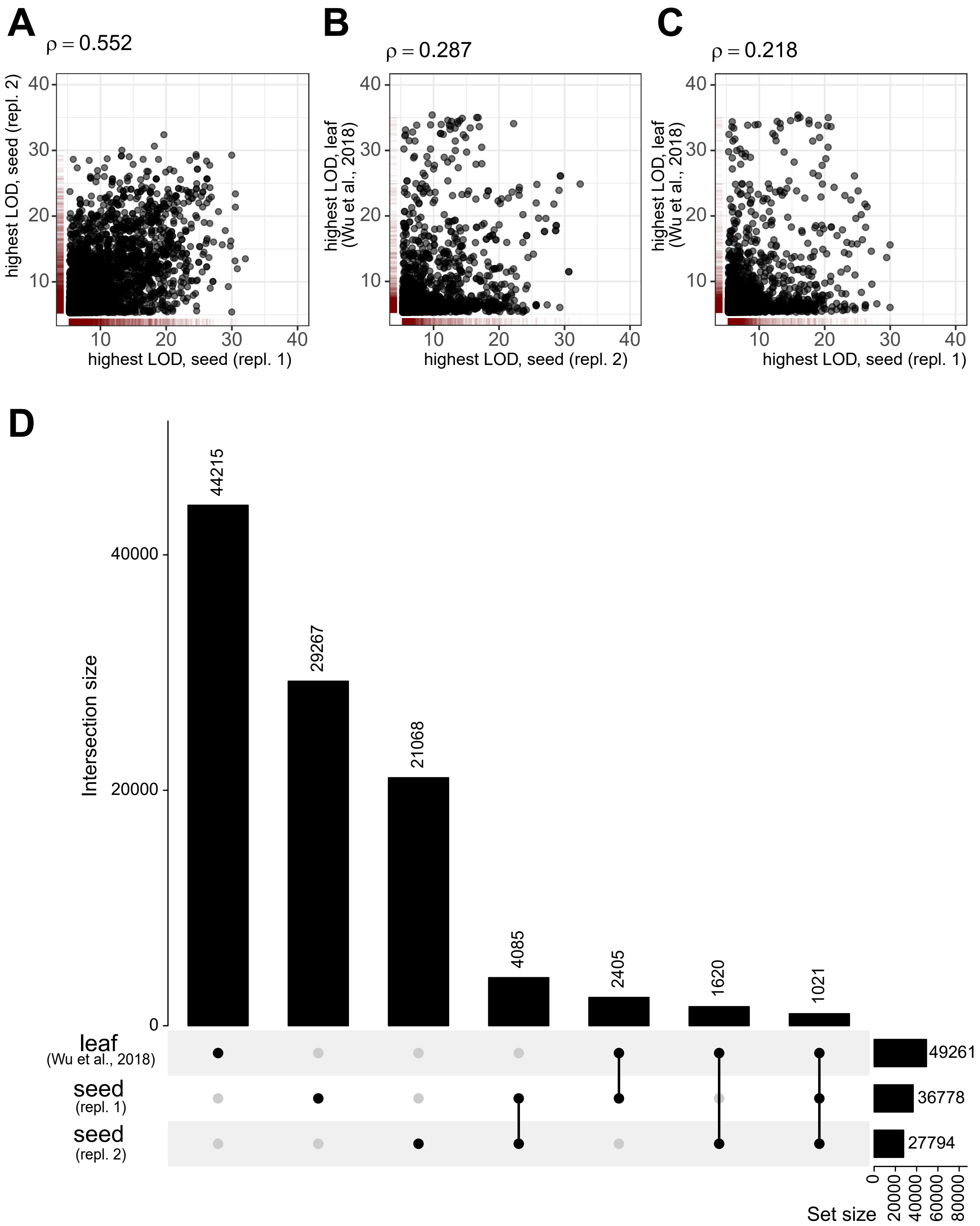

Supplemental Figure S3: Mapping of seed replicates 1 and 2 and leaf$_{\mathrm{Wu}}$ in genome-wide association studies (positive ionization mode). A-C: Scatterplots of highest LOD values of shared QTL per matched mass feature pair for the different datasets. The colored lanes display the density of data points.

The Spearman correlation ρ values are indicated for the different datasets. D: Intersection sets of QTL for mass feature pairs with LOD ≥ 5.3. LOD: logarithm of odds; QTL: quantitative trait loci.

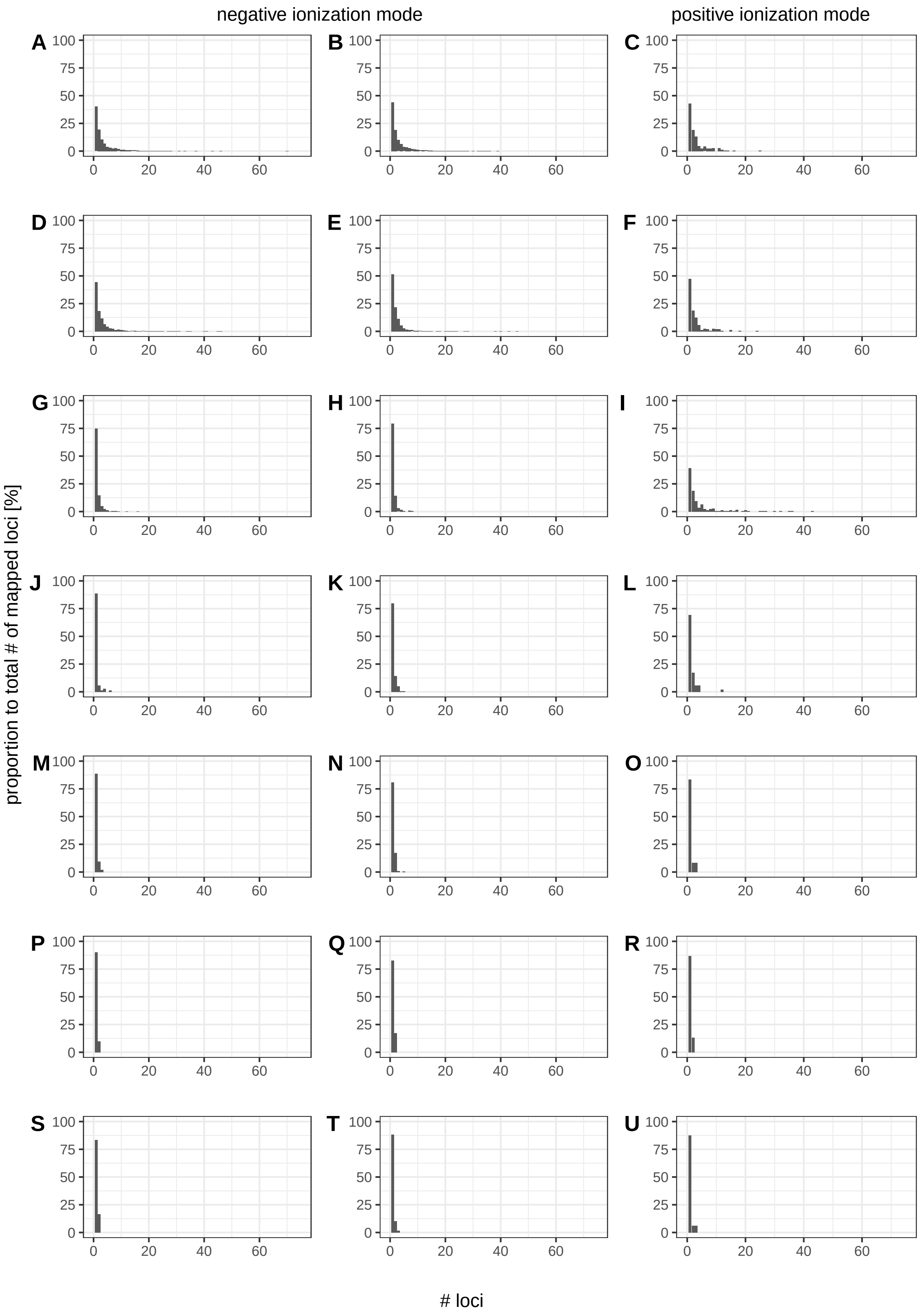

Supplemental Figure S4: Distribution of number of mass features mapped to locus/loci per intersection set. Each panel shows how often a specific mass feature was mapped to a locus/loci per intersection set (e.g., a value of 1 on the x-axis means that this mass feature was mapped to only one locus, a

value of 50% on the y-axis means that 50% of all loci were mapped to one locus if x=1). For the intersection sets seed (repl. 1), seed (repl. 2), leaf_{Wu} and leaf_{Zhu} a higher proportion of mass features was found that were associated with several loci. This can be attributed to random sources of measurement errors, to associations of non-causative markers with a given trait, to linkage with causative markers (Korte and Farlow, 2013), or to environmental effects. The mass features of the other intersection sets show an association with mainly one locus. A: seed (repl. 1). B: seed (repl. 2). C: seed (repl. 1). D: leaf_{Wu}. E: leaf_{Zhu}. F: seed (repl. 2). G: seed (repl. 1) and seed (repl. 2). H: seed (repl. 1) and leaf_{Wu}. I: leaf_{Wu}. J: seed (repl. 1) and leaf_{Zhu}. K: seed (repl. 2) and leaf_{Wu}. L: seed (repl. 1) and seed (repl. 2). M: seed (repl. 2) and leaf_{Zhu}. N: seed (repl. 1) and seed (repl. 2) and leaf_{Wu}. O: seed (repl. 1) and leaf_{Wu}. P: seed (repl. 1) and seed (repl. 2) and leaf_{Zhu}. Q: seed (repl. 1) and leaf_{Wu} and leaf_{Zhu}. R: seed (repl. 2) and leaf_{Wu}. S: seed (repl. 2) and leaf_{Wu} and leaf_{Zhu}. T: seed (repl. 1) and seed (repl. 2) and leaf_{Wu} and leaf_{Zhu}. U: seed (repl. 1) and seed (repl. 2) and leaf_{Wu}.

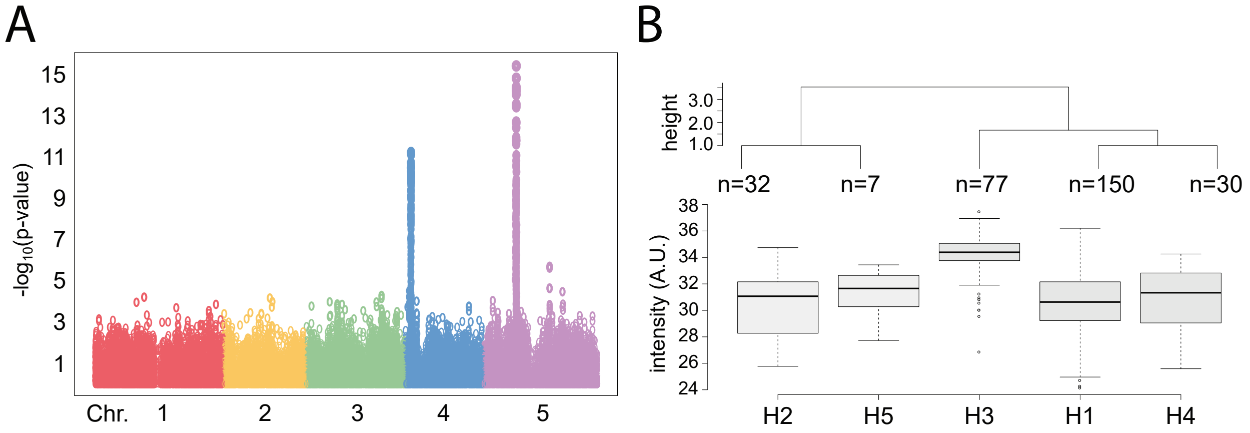

Supplemental Figure S5: Genome-wide association mapping for 3-hydroxypropyl glucosinolate (seed, negative ionization mode). A: The Manhattan plot of 3-hydroxypropyl glucosinolate shows two peaks in each replicate on chromosomes 4 (highest LOD: 11.22) and 5 (15.39). These loci contain the genes *AOP1*, *AOP2*, and *AOP3* (chromosome 4), *MAM1* and *MAM3* (chromosome 5) that are involved in glucosinolate biosynthesis. B: Haplotype analysis of metabolite levels of 3-hydroxypropyl glucosinolate. Shown are the $\log_2$-normalized intensities (A.U.) in boxplots (center line, median; lower and upper box limits, quartiles 1 and 3; whiskers, 1.5x interquartile range; points, outliers). The nucleotide sequence differences were statistically associated with the levels of 3-hydroxypropyl glucosinolate (ANOVA q-value: 7.05e-21 for replicate 2). Only data for seed replicate 2 is shown in A and B. The data for seed replicate 1 is depicted in Figure 2. *AOP*: *alkenyl hydroxyalkyl producing*; A.U.: arbitrary units; LD: linkage disequilibrium; LOD: logarithm of odds; *MAM*: *methylthioalkylmalate synthase*.

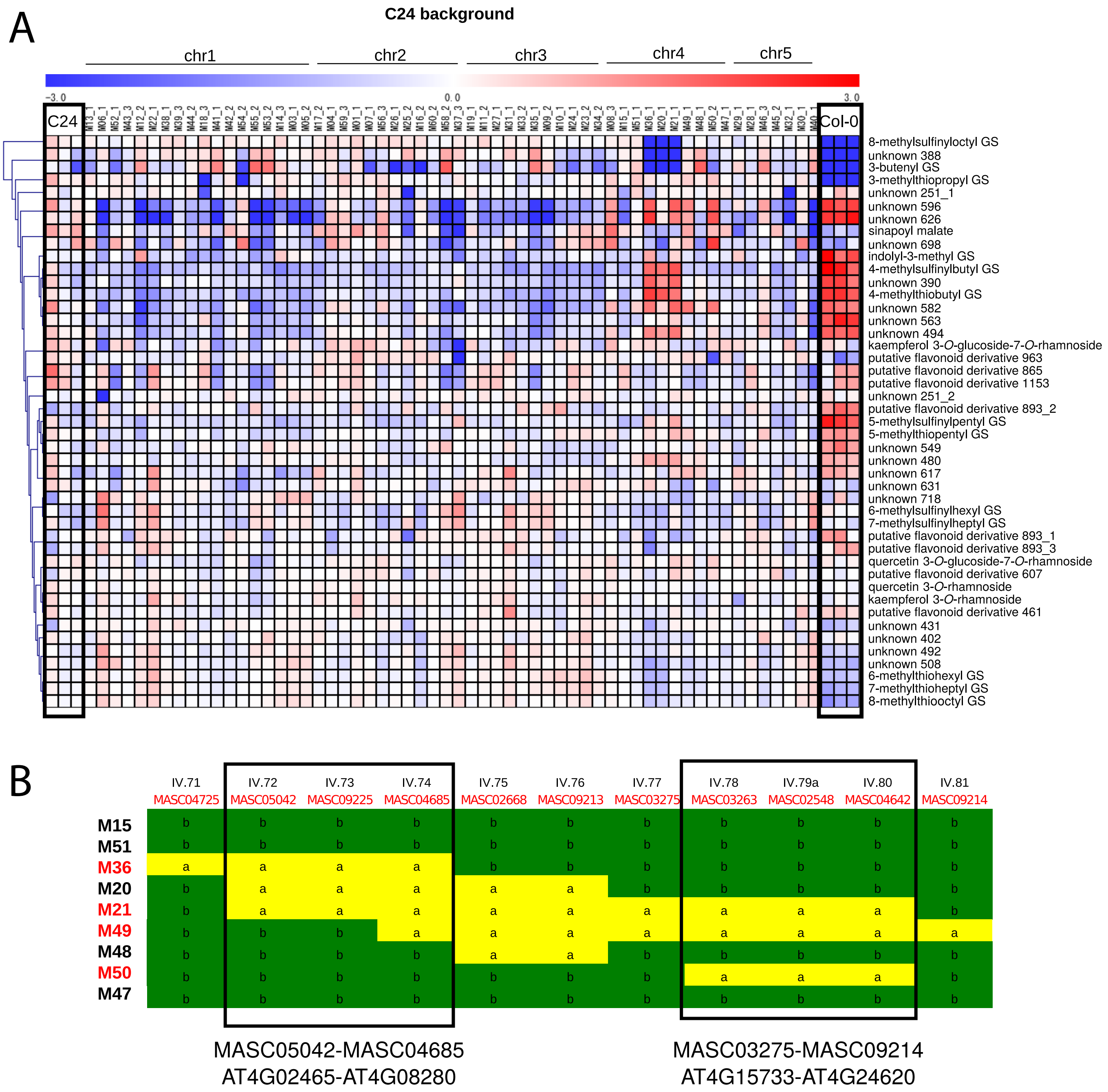

Supplemental Figure S6: QTL mapping using near-isogenic introgression lines between C24 and Col-0 seeds (C24 background). A: Heatmap of relative seed metabolite levels for C24, Col-0 and NILs with C24 background. B: Genomic region for NILs and allele identity based on MASC genomic markers. The highlighted regions refer to the lines with Col-0 alleles that show altered levels of glucosinolates and of the unknowns 596 and 626. A: genomic region from Col-0; b: genomic region from C24; GS: glucosinolates; NIL: near-isogenic line.

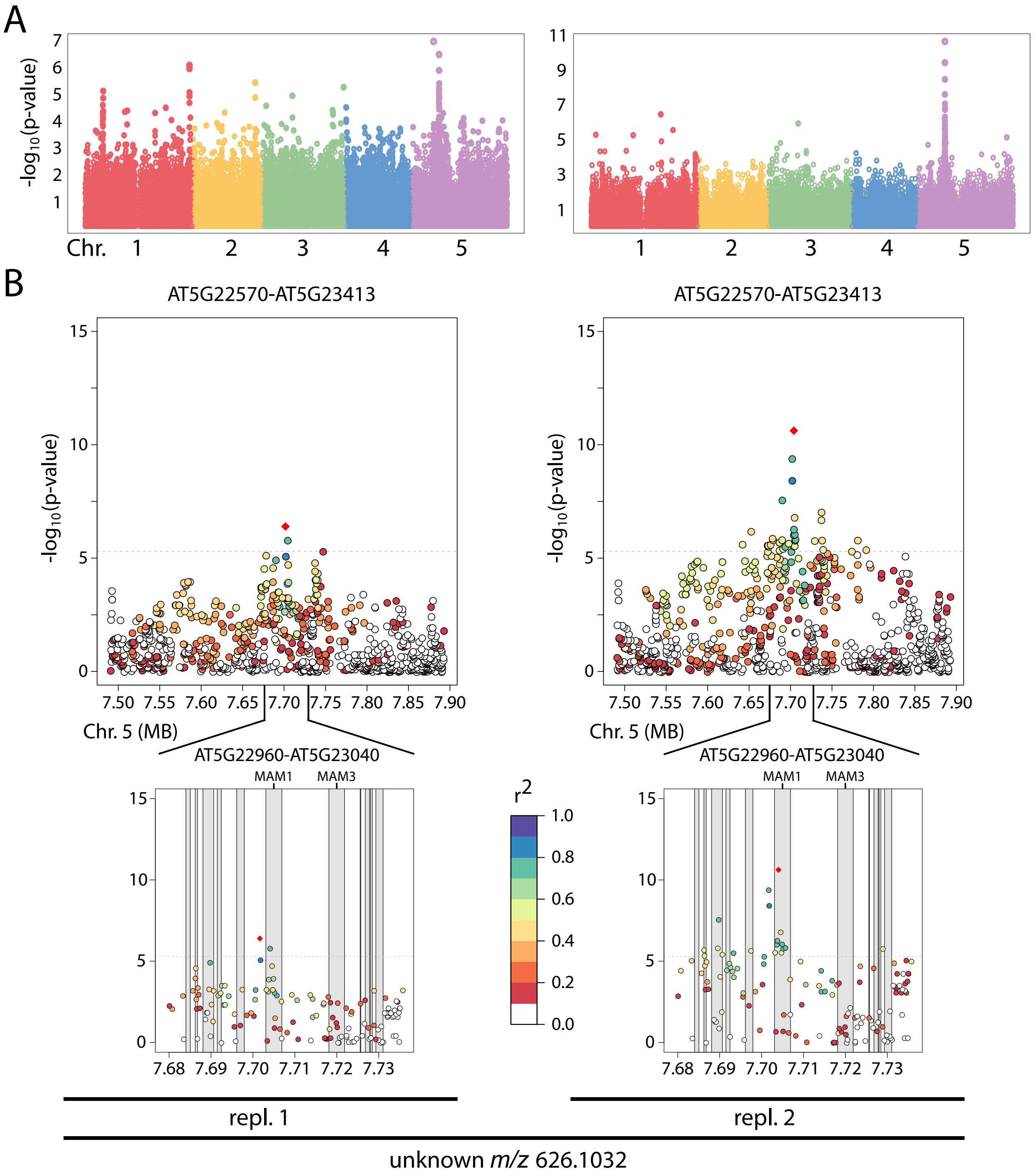

Supplemental Figure S7: Manhattan plot and linkage disequilibrium analysis of the unknown 626 (seed, negative ionization mode). A: Manhattan plot for seed replicate 1 (left) and seed replicate 2 (right). The plots show an association of the unknown 626 with a genomic region on chromosome 5 containing *MAM1* and *MAM3*. B: The highest LOD score is obtained within the region of *MAM1*. The LOD values decay quickly when moving away from *MAM1*. LOD: logarithm of odds; *MAM*: *methylthioalkylmalate synthase*.

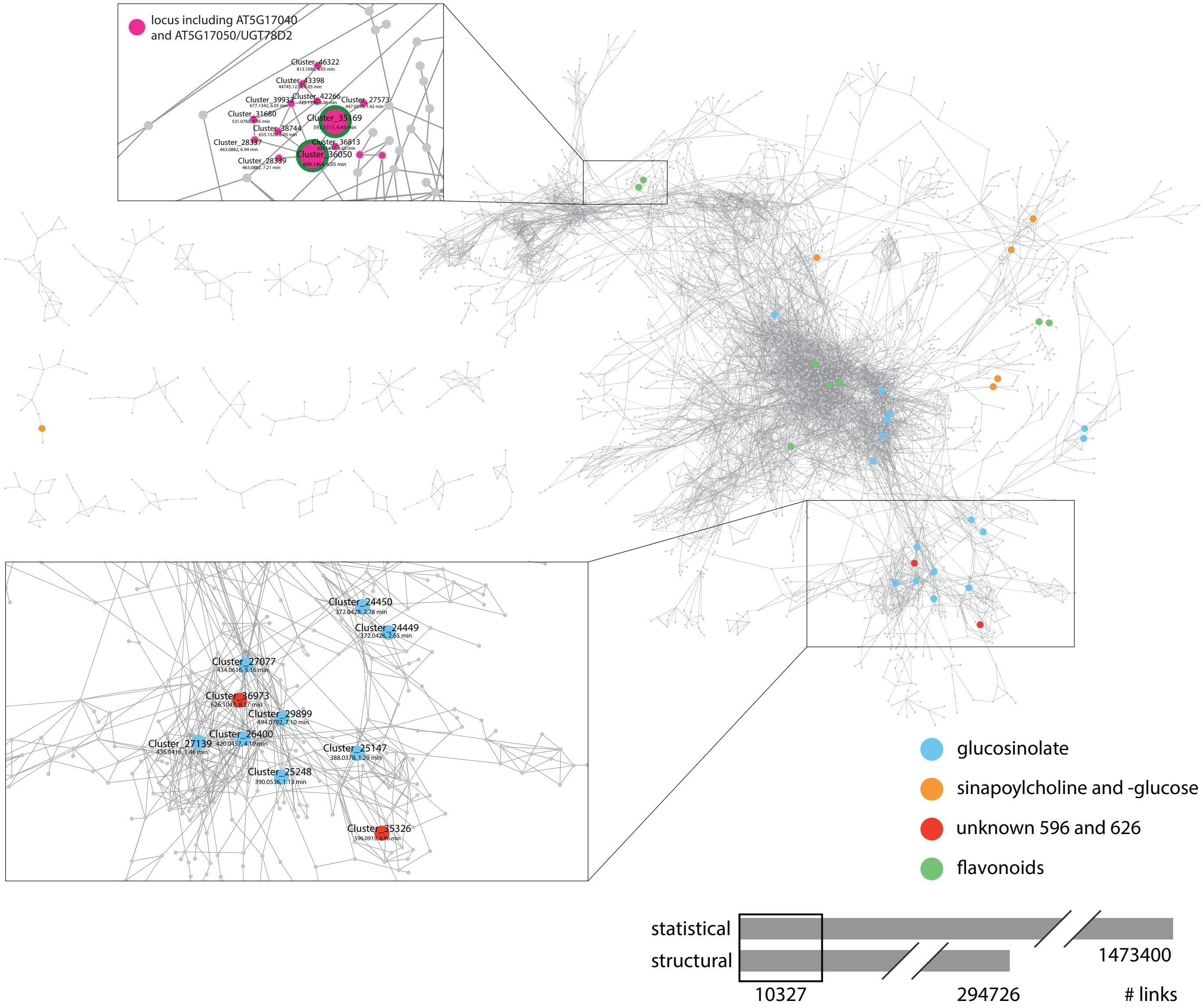

Supplemental Figure S8: Seed metabolite network of mapped mass features (negative ionization mode). The network was constructed by `MetNet` and consists of 8819 mass features (vertices) and 16090 joint edges from the structural and statistical network inference. The 8819 mass features showed associations to at least one locus in one replicate. Mass features corresponding to annotated metabolites are highlighted (glucosinolates, sinapoylcholine and -glucose, unknown 596 and 626, and flavonoids). The figure shows only the major network components. m/z and retention time values are taken from the alignment of mass spectrometric data of replicate 1. Linking mass features to the unknowns 596 and 626 showed association with the *GS-ALK*/*GS-OHP*, and/or *GS-ELONG* loci with a LOD $\geq$ 5.3. First neighbors of the unknown 596 were m/z 390.0329 (retention time: 0.89 min), 390.0329 (1.29, 4-methylthiobutyl GS), 800.1222 (9.3 min). First neighbors of the unknown 626 were m/z 420.0457 (4.10 min), 628.1001 (8.27 min), 640.1201 (8.78 min), 642.0990 (6.42 min), 642.0994 (6.48 min), 648.0857 (8.27 min), 694.0924 (8.27 min). Linking mass features to quercetin-containing flavonols showed association with the locus containing *AT5G17040* and *AT5G17050*/*UGT78D2* with a LOD $\geq$ 5.3. The highlighted mass features also showed associations with other loci (LOD $\geq$ 5.3). LOD: logarithm of odds.

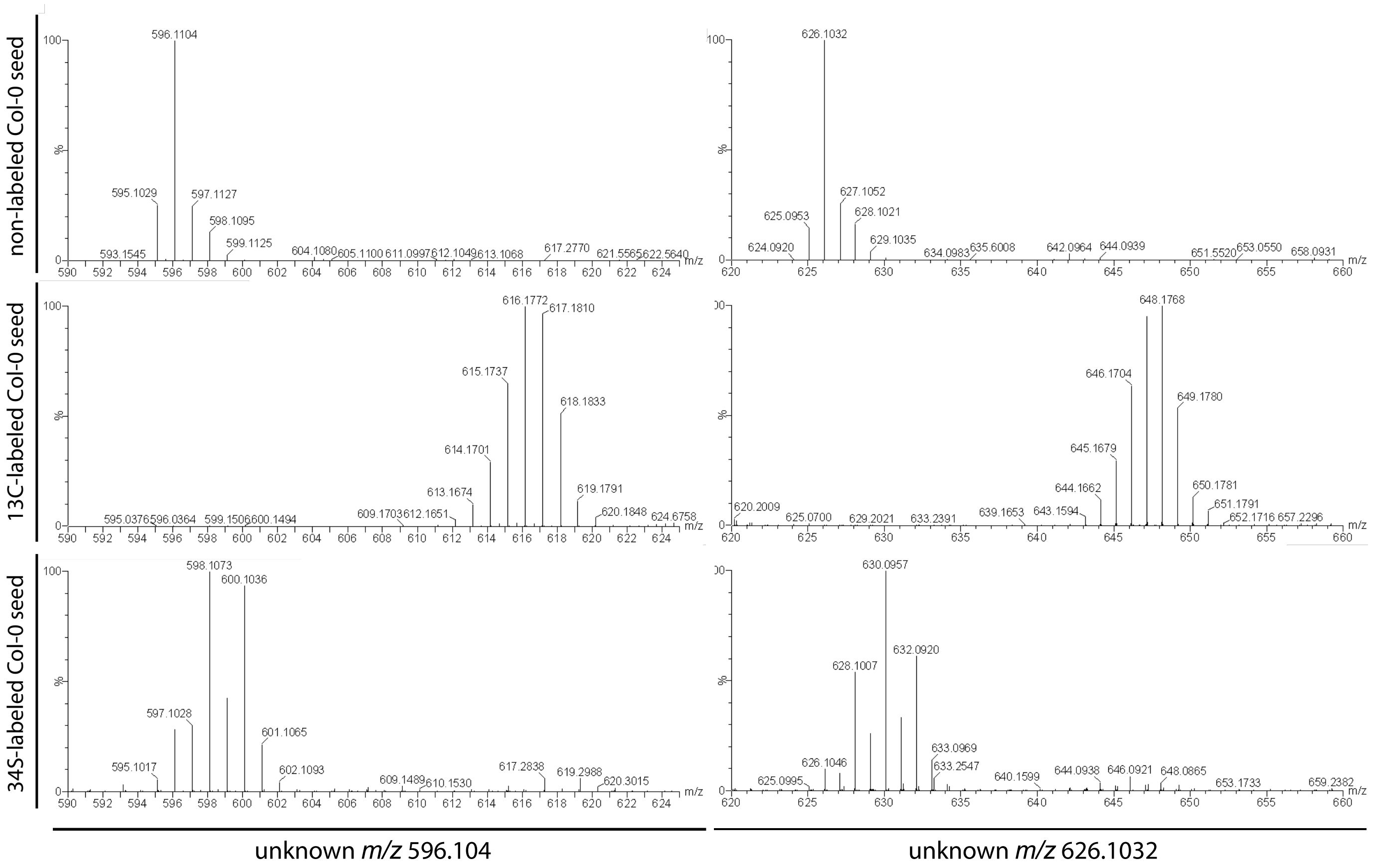

Supplemental Figure S9: Effects of ^{13}C and ^{34}S feeding on the *m/z* of the unknowns 596 and 626 (negative ionization mode). The measurement was performed via LC-QTOF-MS and revealed that the unknown 596 (*m/z* 596.1104) and 626 (*m/z* 626.1032) contains 22 C atoms and 23 C atoms based on isotope feeding with ^{13}C, respectively, and two and three S atoms based on isotope feeding with ^{34}S.

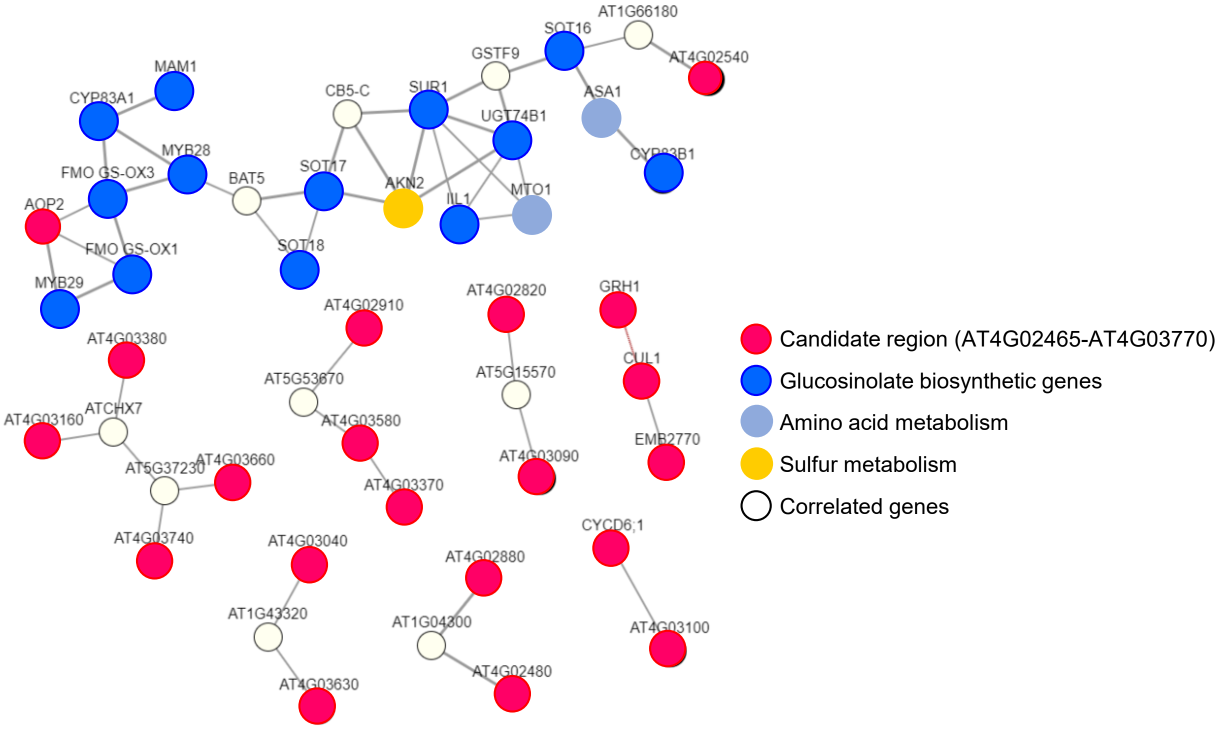

Supplemental Figure S10: Gene co-expression network analysis of glucosinolate biosynthetic genes and genes in the candidate genomics region. Known glucosinolate biosynthetic genes are used in Network drawer https://atted.jp/top_draw/#NetworkDrawer) of Arabidopsis tissue data; *MAM1*, *AT5G23010*; *MAM3*, *AT5G23020*; *CYP79A2*, *AT5G05260*; *CYP83B1|ATR4|SUR2*, *AT4G31500*; *UGT74B1*, *AT1G24000*; *ST*, *AT1G74100*; *BZO1*, *AT1G65880*; *CYP79F1*, *AT1G16410*; *CYP79F2*, *AT1G16400*; *CYP83A1*, *AT4G13770*; *SUR1*, *AT2G20610*; *UGT74B1*, *AT1G24100*; *AtSOT17*, *AT1G18590*; *AtSOT18*, *AT1G74090*; *MYB28*, *AT5G61420*; *MYB29*, *AT5G07690*; *MYB34*, *AT5G60890*; *BGLU30*, *AT3G60140* (Ath-m.c-4-2_tis, "add few genes"). Color of nodes indicate genes located in candidate genomics region (red); glucosinolate biosynthetic genes (blue); amino acid metabolic genes (light blue); sulfur metabolism genes (yellow); correlated genes (white). A RNA-seq profiling dataset (GSE94457) of 10 time points during germination in Arabidopsis seeds can be found in Supplemental Table S14.

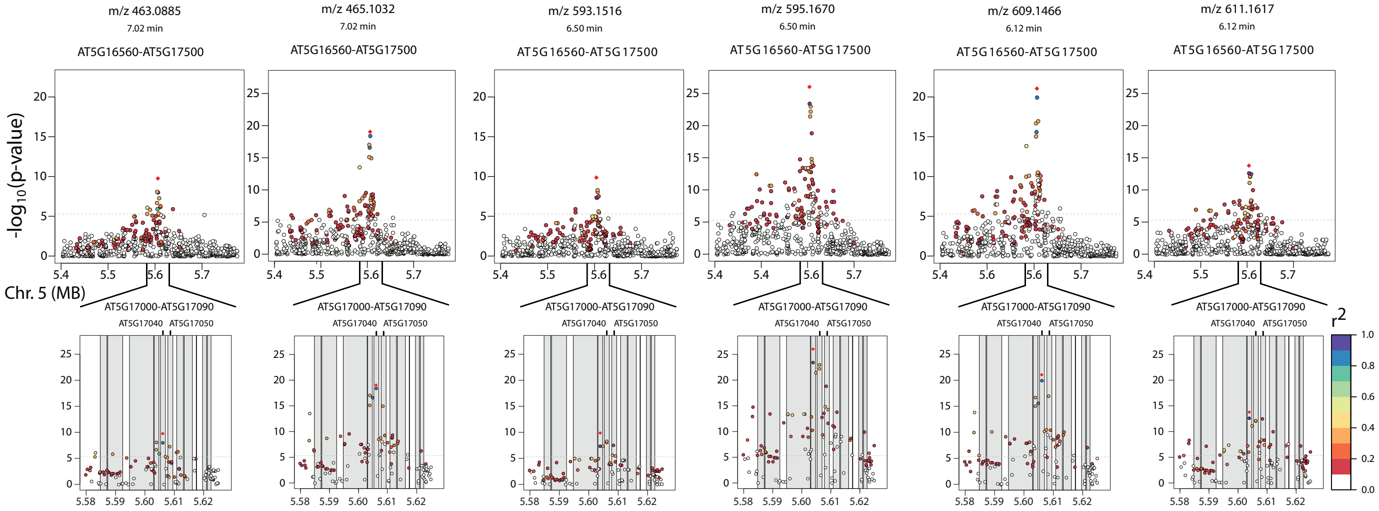

Supplemental Figure S11: Flavonoid biosynthetic pathway: Linkage disequilibrium analysis for quercetin-containing flavonols (seed, negative and positive ionization mode). The highest LOD is achieved for a SNP within the region of *AT5G17030* or *AT5G17040*. Standardized LD r^2 is relatively low for the SNPs that are located within the gene *AT5G17050*. Only data for seed replicate 2 is shown. The data for seed replicate 1 is depicted in Figure 3. LD: linkage disequilibrium; LOD: logarithm of odds; MB: megabase; SNP: single nucleotide polymorphism.

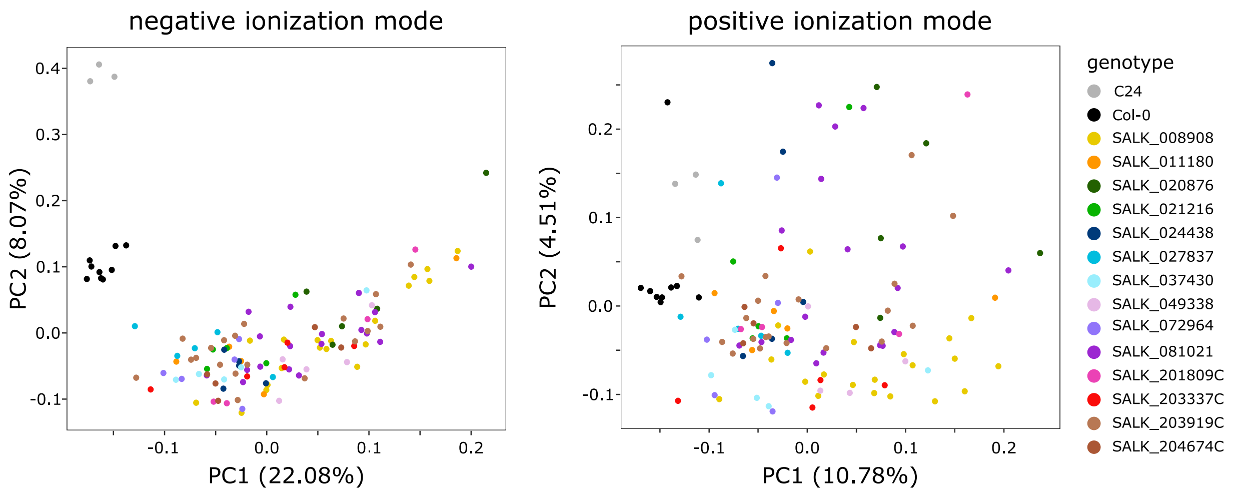

Supplemental Figure S12: Principal component analysis for seed metabolite analysis of SALK lines (replicate 1). Negative ionization mode: The first two PCs explain 30.15% of the variance in the seed dataset. 27716 mass features were used for the PC analysis. The projection of the mass feature levels of C24 (gray dots) and Col-0 (black dots) are located close to each other for each genotype and are distinct from the SALK mutants. Positive ionization mode: The first two PCs explain 15.29% of the variance in the seed dataset. 28750 mass features were used for the PC analysis. The projections of the mass feature levels of C24 (gray dots) and Col-0 (black dots) are located close to each other for each genotype. PC1 and PC2 do not separate C24 and Col-0 from the SALK line mutants. PC: principal component.

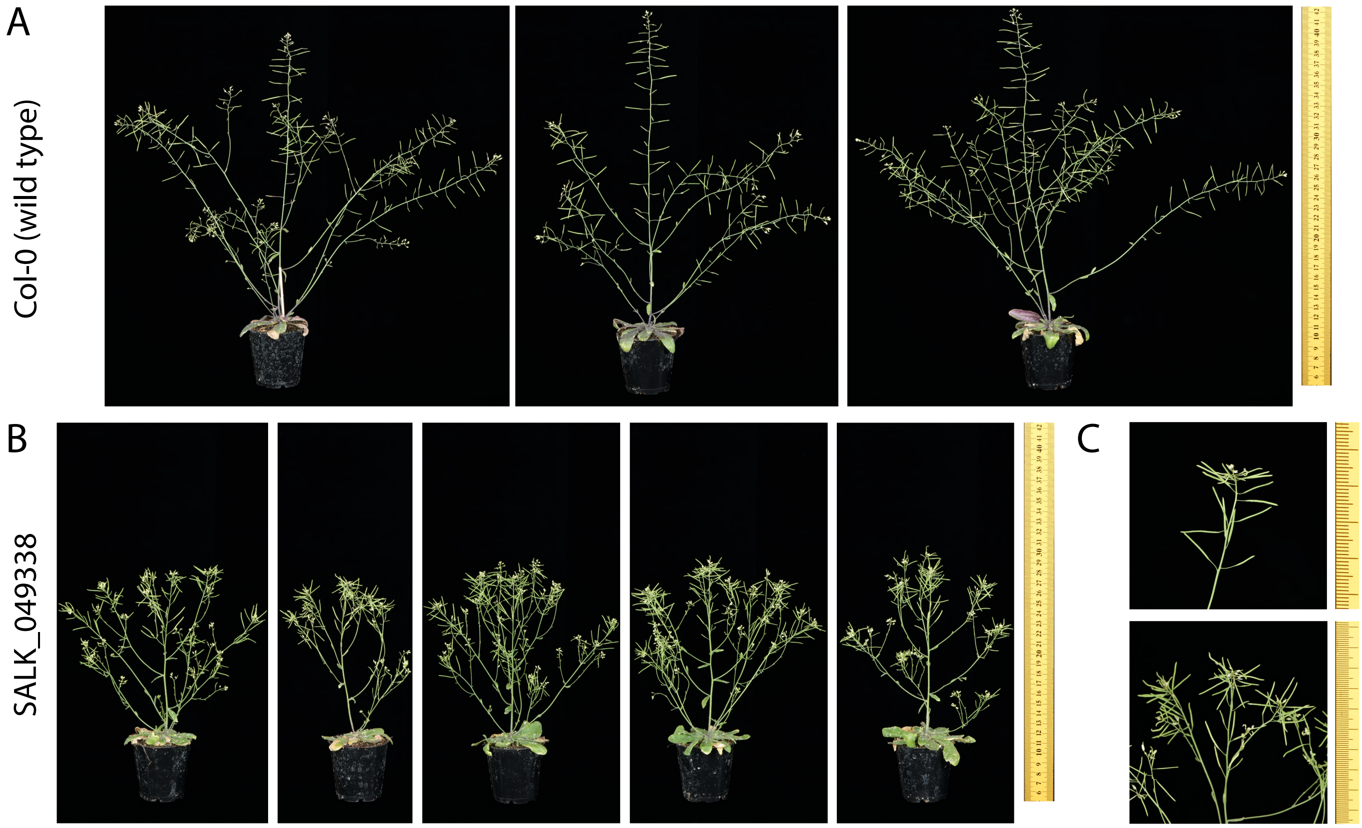

Supplemental Figure S13: Phenotype of Col-0 and SALK_049338 mutant lines. A: Three biological replicates of wild type Col-0. The image on the left is identical to the one shown for the Col-0 wild type in Figure 3B. The scale bar is valid for all images shown here. B: Five biological replicates of SALK_049338 mutant lines, referring to a T-DNA insertion in the exon of *AT5G17050* (*UGT78D2*). The lines exhibited a dwarf phenotype with a loss of apical dominance as reported previously by Yin et al. (2014). The image on the left is identical to the one shown for SALK_049338 in Figure 3B. The scale bar is valid for all images shown here. C: Detail of inflorescence of line SALK_049338. The numerical markings on the scale bar refer to measurements in cm. Distances between marks on the scale bar refer to measurements in mm.
